# Erythroid lineage *Jak2^V617F^* expression promotes atherosclerosis through erythrophagocytosis and macrophage ferroptosis

**DOI:** 10.1172/JCI155724

**Published:** 2022-07-01

**Authors:** Wenli Liu, Nataliya Östberg, Mustafa Yalcinkaya, Huijuan Dou, Kaori Endo-Umeda, Yang Tang, Xintong Hou, Tong Xiao, Trevor P. Fidler, Sandra Abramowicz, Yong-Guang Yang, Oliver Soehnlein, Alan R. Tall, Nan Wang

**Affiliations:** 1 Division of Molecular Medicine, Department of Medicine, Columbia University, New York, New York, USA.; 2 Department of Physiology and Pharmacology (FyFA), Karolinska Institute, Stockholm, Sweden.; 3 Division of Biochemistry, Department of Biomedical Sciences, Nihon University School of Medicine, Tokyo, Japan.; 4 Department of Hematology and; 5 the Key Laboratory of Organ Regeneration and Transplantation of the Ministry of Education, Institute of Immunology, the First Hospital of Jilin University, Changchun, China.; 6 National-local Joint Engineering Laboratory of Animal Models for Human Diseases, Changchun, China.; 7 International Center of Future Science, Jilin University, Changchun, China.; 8 Institute of Experimental Pathology (ExPat), Center of Molecular Biology of Inflammation (ZMBE), University of Münster, Germany.

**Keywords:** Vascular Biology, Atherosclerosis

## Abstract

Elevated hematocrit is associated with cardiovascular risk; however, the causality and mechanisms are unclear. The *JAK2^V617F^* (*Jak2^VF^*) mutation increases cardiovascular risk in myeloproliferative disorders and in clonal hematopoiesis. *Jak2^VF^* mice with elevated WBCs, platelets, and RBCs display accelerated atherosclerosis and macrophage erythrophagocytosis. To investigate whether selective erythroid *Jak2^VF^* expression promotes atherosclerosis, we developed hyperlipidemic erythropoietin receptor Cre mice that express *Jak2^VF^* in the erythroid lineage (VFEpoR mice). VFEpoR mice without elevated blood cell counts showed increased atherosclerotic plaque necrosis, erythrophagocytosis, and ferroptosis. Selective induction of erythrocytosis with low-dose erythropoietin further exacerbated atherosclerosis with prominent ferroptosis, lipid peroxidation, and endothelial damage. VFEpoR RBCs had reduced antioxidant defenses and increased lipid hydroperoxides. Phagocytosis of human or murine WT or JAK2^VF^ RBCs by WT macrophages induced ferroptosis, which was prevented by the ferroptosis inhibitor liproxstatin-1. Liproxstatin-1 reversed increased atherosclerosis, lipid peroxidation, ferroptosis, and endothelial damage in VFEpoR mice and in *Jak2^VF^* chimeric mice simulating clonal hematopoiesis, but had no impact in controls. Erythroid lineage *Jak2^VF^* expression led to qualitative and quantitative defects in RBCs that exacerbated atherosclerosis. Phagocytosis of RBCs by plaque macrophages promoted ferroptosis, suggesting a therapeutic target for reducing RBC-mediated cardiovascular risk.

## Graphical abstract

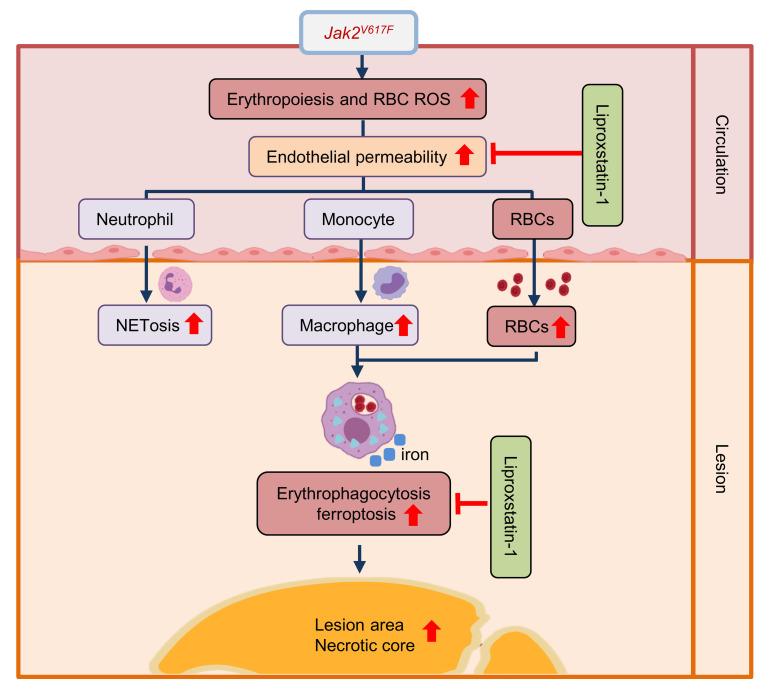

## Introduction

Elevated hematocrit is associated with increased atherothrombotic cardiovascular disease (ACD), the number one cause of death and disability in Western societies (1, 2). Observational studies have shown that human coronary atherosclerotic lesions contain RBCs, RBC debris, and iron, and that RBC infiltration and macrophage erythrophagocytosis are associated with lesional lipid and protein oxidation (3–5). RBC fragments are more evident in unstable atherosclerotic plaques with large necrotic cores and thin fibrous caps, suggesting that RBCs may serve as a source of cholesterol and oxidized lipids that promote formation of unstable atherosclerotic plaques (3, 4). While these observations are suggestive, the causal role of RBCs in the development and progression of atherosclerosis remains uncertain, due in part to the concomitant infiltration and activation of inflammatory cells that have a well-established role in promoting ACD (6–8).

JAK2^VF^, a gain-of-function mutation that is a common driver of myeloproliferative disorders, such as polycythemia vera and essential thrombocytosis, is prominently associated with ACD (9, 10). While JAK2^VF^-driven myeloproliferative disorders are associated with increased hematocrit, platelet and leukocyte counts, mutations in JAK2 exon 12 have been identified in patients with myeloproliferative disorders who have isolated erythrocytosis (9) and increased atherothrombotic events (11). The JAK2^VF^ variant is also associated with clonal hematopoiesis, a common condition in the elderly that increases the risk of atherothrombotic disease, including early onset myocardial infarction (12). Mouse models of clonal hematopoiesis have shown increased atherosclerosis (13–15) and heart failure (16, 17). The JAK2^VF^ mutation, which is found in 3.1% of the general European population (18), occurs at a younger age and increases coronary artery disease (12) and venous thromboembolic disease (19, 20) more prominently than the other clonal hematopoiesis variants.

We have shown that hematopoietic *Jak2^VF^* expression increases ACD despite lowering LDL cholesterol in both mice and humans (21, 22). Mice that express *Jak2^VF^* in multiple hematopoietic cell lineages have elevated RBCs, platelets, and WBCs; prominent lesional necrotic core formation; and inflammatory cell infiltration (22). Striking RBC accumulation and iron deposits as well as colocalization of RBC and macrophage markers suggest erythrophagocytosis within atherosclerotic lesions. Recent studies have shown that erythrophagocytosis can lead to ferroptotic cell death of splenic red pulp macrophages in mice transfused with aged RBCs (23). Ferroptosis is a form of caspase-independent cell death promoted by Fe^2+^ and phospholipid hydroperoxides (22) that could potentially promote atherosclerosis in *Jak2^VF^* mice.

To investigate whether RBCs contribute to atherogenesis independently from leukocytes or platelets, we developed hyperlipidemic mice with selective expression of *Jak2^VF^* in the erythroid lineage using erythropoietin receptor Cre (24), referred to as VFEpoR mice. These mice displayed increased RBC and Fe staining in plaques and increased plaque necrosis. Low-dose erythropoietin (EPO) injection induced erythrocytosis without concomitant leukocytosis or thrombocytosis and further exacerbated plaque necrosis. We used a marker of ferroptosis (25) to show prominent ferroptosis in atherosclerotic lesions of mice. Moreover, inhibition of ferroptosis with liproxstatin-1 (Liprox-1) markedly reduced lesion area and necrosis in VFEpoR mice, including in a clonal hematopoiesis model, but not in control hyperlipidemic mice. Thus, we have established an independent role of *Jak2^VF^* erythroid lineage expression in the exacerbation of atherosclerosis and have identified ferroptosis as a potential therapeutic target in ACD.

## Results

### Erythroid lineage–specific expression of Jak2^VF^ promotes features of plaque instability in VFEpoR mice.

**Figure 1:**
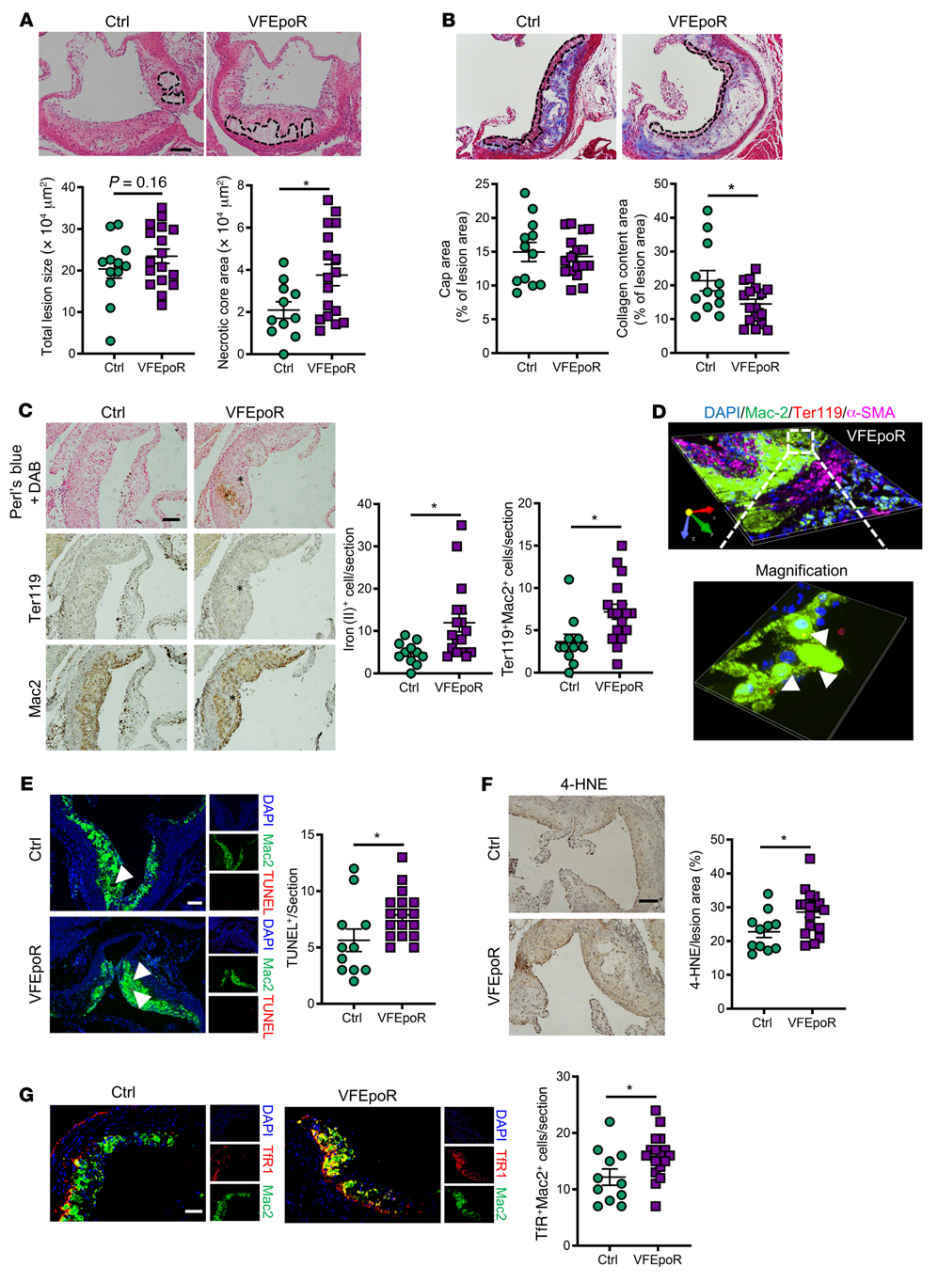
Characterization of atherosclerotic plaques of VFEpoR mice. Control (EpoR-Cre) and VFEpoR mice were fed a Western diet and treated with low-density lipoprotein receptor antisense oligonucleotides (ASO) weekly for 12 weeks. (**A**) H&E staining and quantification of total lesion area and necrotic core area in the aortic root. Necrotic core regions are indicated by broken lines. Scale bar: 100 μm. (**B**) Aortic root sections were stained with Masson’s trichrome staining for fibrous cap (red, outlined by broken lines) and collagen (blue) content area, and then quantified as the ratio of total lesion area. Scale bar: 100 μm. (**C**) Redox-active iron deposition (Perl’s blue + DAB); IHC staining of RBCs (anti-Ter119) and macrophages (anti-Mac2) in aortic roots. Bar graph shows quantification of iron (II)–positive and erythrophagocytosis (Ter119^+^Mac2^+^) cell counts per section. Scale bar: 100 μm. (**D**) Representative 3D-rendered image from VFEpoR mice aortic root lesions of macrophage (anti-Mac2, green), smooth muscle cell (ACTA-2, magenta), and RBCs (anti-Ter119, red). Size: 332.80 μm × 332.80 μm × 7.50 μm. Calibration: XY:0.33 μm, Z:1.50 μm. Arrowheads show macrophage erythrophagocytosis. (**E**) TUNEL and immunofluorescence staining of macrophage (anti-Mac2) in aortic roots and quantification of TUNEL-positive cell counts per section. Scale bar: 75 μm. (**F**) Lipid peroxidation product 4-HNE staining, quantified as the percentage of total lesion area. Scale bar: 100 μm. (**G**) Immunofluorescence staining of TfR1 and macrophage (anti-Mac2), and quantification of TfR1 and macrophage costaining cell counts per section. Scale bar: 50 μm. Unpaired 2-tailed *t* test or Mann–Whitney *U* test, *P < 0.05.

To restrict the expression of *Jak2^VF^* to the erythroid lineage, we used EpoR-Cre mice to create a conditional knockin of *Jak2^VF^*. Allele-specific qPCR analysis showed that *Jak2^VF^* expression was restricted to erythroid progenitor and precursor cells (Supplemental Figure 1A; supplemental material available online with this article; https://doi.org/10.1172/JCI155724DS1), leading to a modest increase in megakaryocyte-erythroid progenitors and no significant change of hematocrit (Supplemental Figure 1B). EpoR-Cre did not induce *Jak2^VF^* expression in aortic endothelial cells, myeloid cells, or the heart (Supplemental Figure 1C). We monitored blood cell counts up to 11 months of age for the VFEpoR and littermate control EpoR mice and did not observe changes in hematocrit or RBC, WBC, and platelet counts in the VFEpoR mice, but we observed significantly increased red cell distribution width (RDW) over time (Supplemental Figure 1D), a known marker of CVD risk (26). We were unable to generate VFEpoR/*Ldlr^–/–^* mice by cross-breeding because of linkage disequilibrium between the *Epor* and *Ldlr* genes. As an alternative, we used *Ldlr* antisense oligonucleotide (ASO; ref. 27) to knock down *Ldlr* in VFEpoR mice. After 12 weeks of Western diet (WD) feeding, VFEpoR and littermate EpoR-Cre control mice had comparable plasma cholesterol levels, body weight, spleen weight, RBC counts, hematocrit, WBC, and platelet counts (Supplemental Figure 1, E–H). VFEpoR mice displayed significantly increased RDW (Supplemental Figure 1G). Although overall lesion area was not increased in VFEpoR mice, they displayed an increase in necrotic core area and lower overall collagen content (Figure 1, A and B), without a change in fibrous cap area (Figure 1B). We then characterized atherosclerosis in more advanced lesions after WD feeding for 22 weeks. VFEpoR and control mice again showed comparable plasma cholesterol levels, body weight, spleen weight, RBCs, hematocrit, WBCs, and platelet counts (Supplemental Figure 1, I–K). VFEpoR mice also showed increased RDW and lower serum EPO level (Supplemental Figure 1, J and L), similar to patients with JAK2^VF^ myeloproliferative neoplasm (28). Total aortic root lesion area showed no change (Supplemental Figure 1M). However, features of plaque instability, including increased necrotic core area, decreased fibrous cap area, and reduced lesional collagen content, were all prominent in VFEpoR mice (Supplemental Figure 1, M and N). Costaining of RBC and macrophage markers indicated increased erythrophagocytosis in VFEpoR compared with control mice (Figure 1C). Localization of RBCs inside macrophages was verified by 3D confocal immunofluorescence imaging in VFEpoR mice (Figure 1D). Excessive erythrophagocytosis can liberate non-transferrin-bound iron (29). Accordingly, Perl’s blue plus diaminobenzidine (DAB) staining revealed increased reactive iron deposits in VFEpoR mice (Figure 1C), in a similar distribution to erythrophagocytotic macrophages (Figure 1C). Aortic root lesions of VFEpoR mice had more TUNEL-positive macrophages, indicating increased macrophage death and consistent with the increased necrotic cores (Figure 1E). There was also evidence of increased lipid peroxidation in plaques as shown by increased 4-hydroxynonenal (4-HNE) staining (Figure 1F). Iron deposition and 4-HNE staining in tissues are markers of ferroptosis (30). Recently, a screen of mAbs produced in response to ferroptotic membranes uncovered increased transferrin receptor (TfR) staining as a highly characteristic cellular marker of ferroptosis (25). We found increased TfR staining in atherosclerotic lesions of VFEpoR mice relative to the control mice that was primarily colocalized with macrophages (Figure 1G), indicating macrophage ferroptosis. These findings suggest that even in the absence of erythrocytosis, increased erythrophagocytosis in VFEpoR mice leads to ferroptotic cell death that could contribute to increased necrotic core formation.

### Jak2^VF^-induced erythrocytosis further aggravates atherosclerosis.

Prominent increases in RBCs as well as increased WBCs and platelets are associated with increased risk of atherosclerosis in JAK2^VF^-driven polycythemia vera (22). Moreover, a recent study has linked clonal hematopoiesis mutations to erythrocytosis and increased ACD in a general population, with JAK2^VF^ as a prominent contributor (31). We found that low-dose EPO administration selectively induced increased RBC counts, hematocrit, and RDW but not WBC and platelet counts in VFEpoR mice relative to EpoR-Cre control and WT mice over 12 weeks (Supplemental Figure 2A). To evaluate the atherogenic role of VFEpoR RBCs in a model that mimics human polycythemia, we injected the same low-dose EPO into hyperlipidemic littermate EpoR-Cre control and VFEpoR mice that were fed the WD for 12 weeks. The phenotypes induced by low-dose EPO in hyperlipidemic VFEpoR mice resembled those seen in human polycythemia, with increased RBC counts, hematocrit, RDW, and splenomegaly (Supplemental Figure 2B). However, there were no changes in WBC or platelet counts compared with control mice (Supplemental Figure 2C). Body weight and plasma cholesterol levels were comparable in these 2 groups (Supplemental Figure 2, D and E) throughout the study.

**Figure 2:**
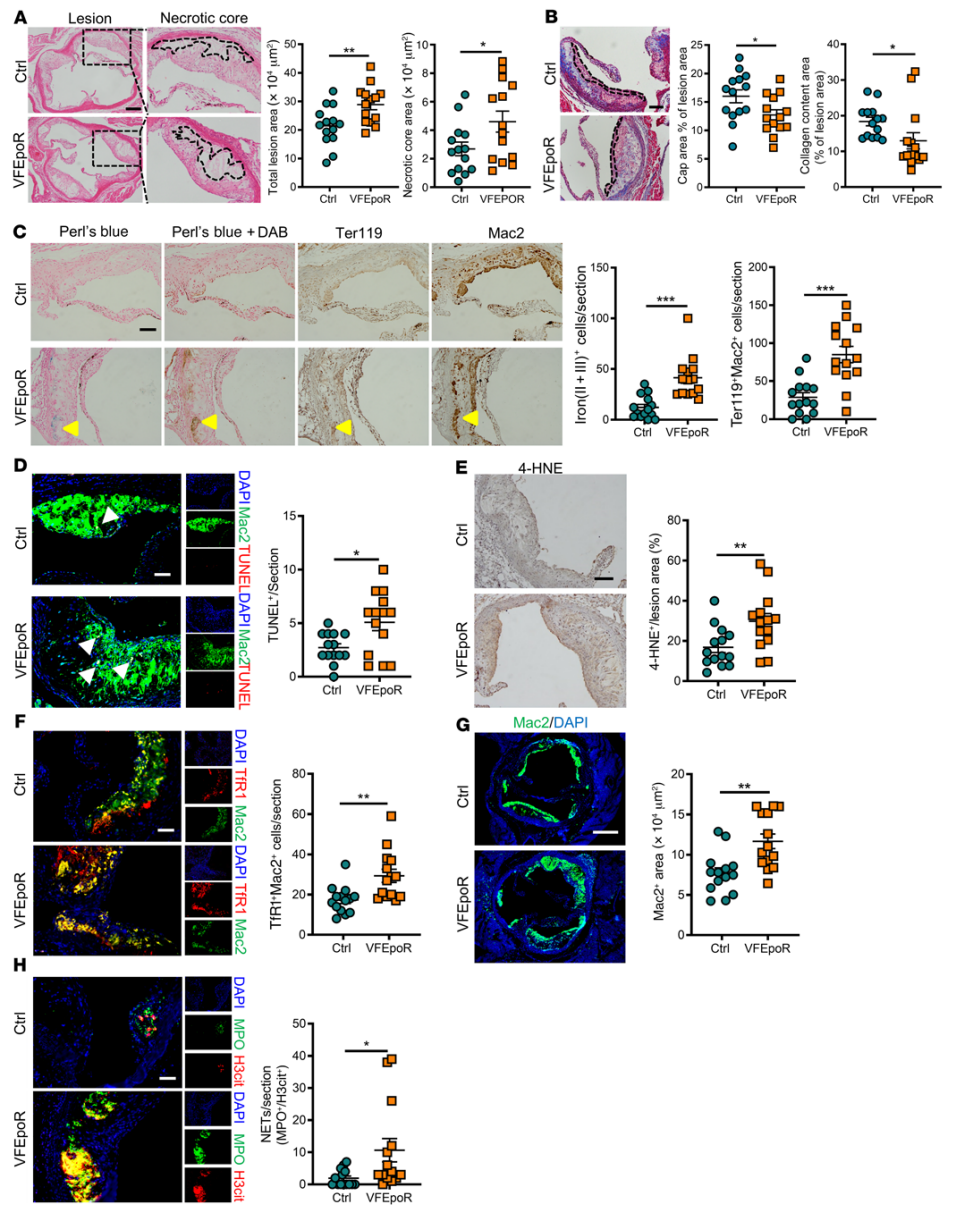
Increased lesion area, plaque instability, and iron deposition in VFEpoR mice after EPO injection. Control and VFEpoR mice were fed a Western diet and treated with LDLR ASO and EPO (3 times per week) for 12 weeks. (**A**) H&E staining of aortic root sections and quantification of absolute lesion and necrotic core area. Necrotic core regions indicated by broken lines. Scale bar: 200 μm. (**B**) Aortic root sections were stained with Masson’s trichrome staining for fibrous cap (red, outlined by broken lines) and collagen (blue) content area, and then quantified as the ratio of total lesion area. Scale bar: 100 μm. (**C**) Iron (Perl’s blue) and redox-active iron deposition (Perl’s blue + DAB), IHC staining of RBCs (anti-Ter119) and macrophages (anti-Mac2) in aortic roots. Bar graph shows quantification of iron (II + III)–positive and erythrophagocytosis (Ter119^+^Mac2^+^) cell counts per section. Scale bar: 100 μm. (**D**) TUNEL and immunofluorescence staining of macrophage (anti-Mac2) in aortic roots and quantification of TUNEL-positive cell counts per section. Scale bar: 50 μm. (**E**) Lipid peroxidation product 4-HNE staining, quantified as the percentage of total lesion area. Scale bar: 100 μm. (**F**) Immunofluorescence staining of TfR1 and macrophage (anti-Mac2), and quantification of TfR1 and macrophage costaining cell counts per section. Scale bar: 50 μm. (**G**) Aortic root sections were immunostained for Mac2 and quantified as absolute Mac2-positive area. Scale bar: 250 μm. (**H**) Lesions were stained for citrullinated histones (H3Cit) and activated neutrophils using myeloperoxidase (MPO); the overlap H3cit and MPO (NETs) cell counts were quantified. Scale bar: 50 μm. Unpaired 2-tailed *t* test or Mann-Whitney *U* test, *P < 0.05, **P < 0.01, ***P < 0.001.

Total lesion area and necrotic core area of aortic root plaques were significantly increased, whereas fibrous cap area and lesional collagen content were reduced in EPO-injected hyperlipidemic VFEpoR mice compared with control mice (Figure 2, A and B). Likewise, direct visualization of aortic arch lesions showed prominently increased lesion formation along the lesser curvature of the aorta and at aortic branch points that are regions of disturbed blood flow (Supplemental Figure 2F). Consistent with the more severe atherosclerosis phenotypes, there was more prominent staining for lesional RBCs, iron, TUNEL, 4-HNE, and macrophage TfR in VFEpoR mice injected with EPO compared with VFEpoR mice not injected with EPO (Figure 2, C–F). Immunostaining of Mac2 showed increased Mac2-positive aortic root lesion area (Figure 2G) without altered lesional macrophage proliferation (Supplemental Figure 2G) in VFEpoR mice, consistent with increased monocyte entry into plaques and monocyte-to-macrophage differentiation (32). VFEpoR lesions showed increased staining of H3cit, a marker of citrullinated histone, which was largely colocalized with MPO immunostaining, suggesting increased neutrophil extracellular traps (NETs), although lower level MPO could be expressed in macrophages (Figure 2H). Together, these results suggest increased entry not only of RBCs but also of WBCs into plaques, leading to increased macrophage burden and NETosis in addition to macrophage ferroptosis and lipid peroxidation products in VFEpoR mice.

### Increased ROS and lipid peroxidation in VFEpoR RBCs.

**Figure 3:**
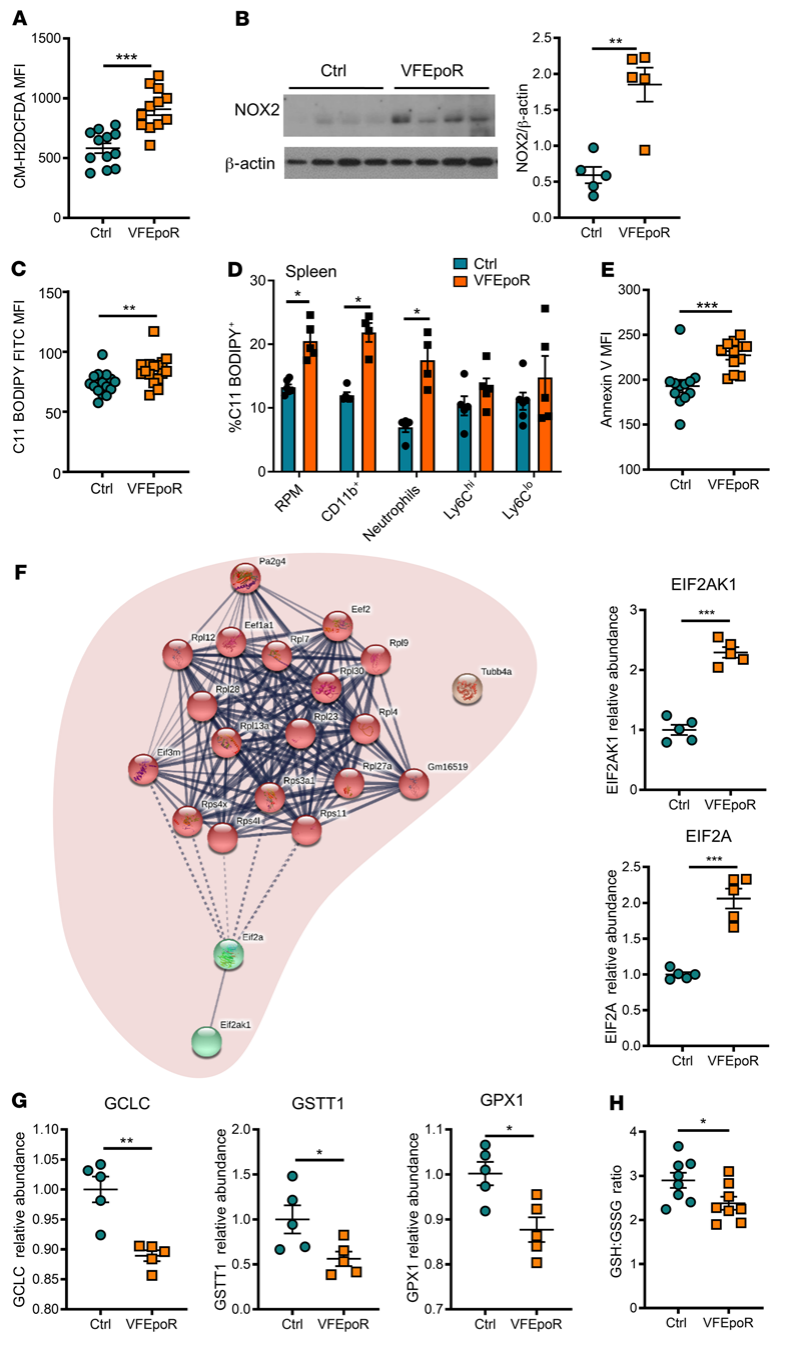
VFEpoR RBCs, red pulp macrophages, and splenic CD11b^+^ cells show increased ROS and lipid peroxidation. Control and VFEpoR mice were fed a Western diet and treated with LDLR ASO and EPO (3 times per week) for 12 weeks. (**A**) ROS in RBCs was assessed by H_2_ DCFDA staining and analyzed by flow cytometry as MFI. (**B**) Immunoblot and quantification of NOX2 expression in RBC lysates, which was normalized to β-actin. (**C**) RBC lipid peroxidation was assessed by C11 BODIPY staining and quantified by flow cytometry as MFI. (**D**) Splenic red pulp macrophages, CD11b^+^ cells, neutrophils, and Ly6C^hi^ and Ly6C^lo^ monocytes were stained with C11 BODIPY for lipid peroxidation and analyzed by flow cytometry. (**E**) Assessment of externalized phosphatidylserine level by annexin V staining in RBCs through flow cytometry. (**F**) Interaction network and clustering of upregulated proteins in VFEpoR RBCs. Bar graphs show relative abundance protein level of EIF2AK1 (also known as heme-regulated inhibitor, HRI) and EIF2A (eukaryotic translation initiation factor 2A). The lines between proteins represent interactions among proteins with confidence level; dashed line is the lowest and thick line is the highest confidence. (**G**) Proteins involved in cellular oxidant stress were analyzed and quantifications of relative abundance of glutamate cysteine ligase catalytic subunit (GCLC), glutathione transferase theta 1 (GSTT1), glutathione peroxidase 1 (GPX1) are shown. (**H**) The level of reduced and oxidized glutathione ratio in RBCs. Unpaired 2-tailed *t* test, *P < 0.05, **P < 0.01, ***P < 0.001.

The infiltration of RBCs into plaques along with evidence of erythrophagocytosis and lipid peroxidation suggested that VFEpoR RBCs might contain increased lipid peroxides. VFEpoR RBCs had elevated levels of ROS as assessed by staining with H_2_ DCFDA, regardless of EPO supplementation (Figure 3A and Supplemental Figure 3A). In addition to nonenzymatic production of ROS by Hb auto-oxidation and iron-mediated Fenton chemistry (33), enzymatic ROS production could also contribute to RBC ROS generation (34). Indeed, NADPH oxidase 2 (NOX2) levels were increased approximately 3-fold in VFEpoR RBCs (Figure 3B). Consistently, lipid peroxidation, as assessed with C11-BODIPY staining, was increased in VFEpoR RBCs (Figure 3C and Supplemental Figure 3B). Cellular lipid peroxidation was also increased in splenic red pulp macrophages and CD11b^+^ myeloid cells, especially neutrophils in VFEpoR mice, consistent with phagocytic uptake of RBCs containing increased lipid peroxides in the spleen (Figure 3D). VFEpoR RBCs also showed increased annexin V staining, which has been associated with aging changes in RBCs (ref. 35 and Figure 3E). Together, these observations suggest increased ROS and lipid peroxidation that may lead to premature aging of VFEpoR RBCs, potentially seeding lipid peroxidation in splenic and lesional WBCs and promoting atherosclerosis.

### Proteomics analysis indicates reduced antioxidant defenses in VFEpoR RBCs.

To explore underlying defects promoting oxidative changes in *Jak2^VF^* RBCs, we conducted unbiased, high-throughput proteomics profiling. This showed a prominent increase in reticulocyte proteins consistent with reticulocytosis in VFEpoR mice (Supplemental Figure 3C). However, reticulocytes did not show an increase in ROS (Supplemental Figure 3D). There was an increase in EIF2AK1 (also known as heme-regulated inhibitor, HRI) and EIF2A (Figure 3F), consistent with increased oxidative stress (36). Notably, there were decreased levels of several key enzymes in glutathione metabolism in VFEpoR RBCs, including glutamate cysteine ligase catalytic subunit (GCLC), the rate-limiting enzyme in GSH synthesis (37, 38); glutathione s-transferase theta 1 (GSTT1), involved in conjugating GSH to hydrophobic substrates (39), consistent with a previous report (40); and glutathione peroxidase 1 (GPX1), the major isoform of GPX in RBCs (41) that utilizes GSH to reduce lipid hydroperoxides (Figure 3G). The decreased expression of these genes involved in glutathione metabolism was associated with a reduced glutathione to oxidized glutathione ratio in VFEpoR RBCs (Figure 3H). Together, these data confirmed increased oxidative stress in VFEpoR RBCs and uncovered decreased antioxidant activity involving protective glutathione pathways that reduce lipid peroxidation.

### Increased arterial endothelial permeability in VFEpoR mice.

**Figure 4:**
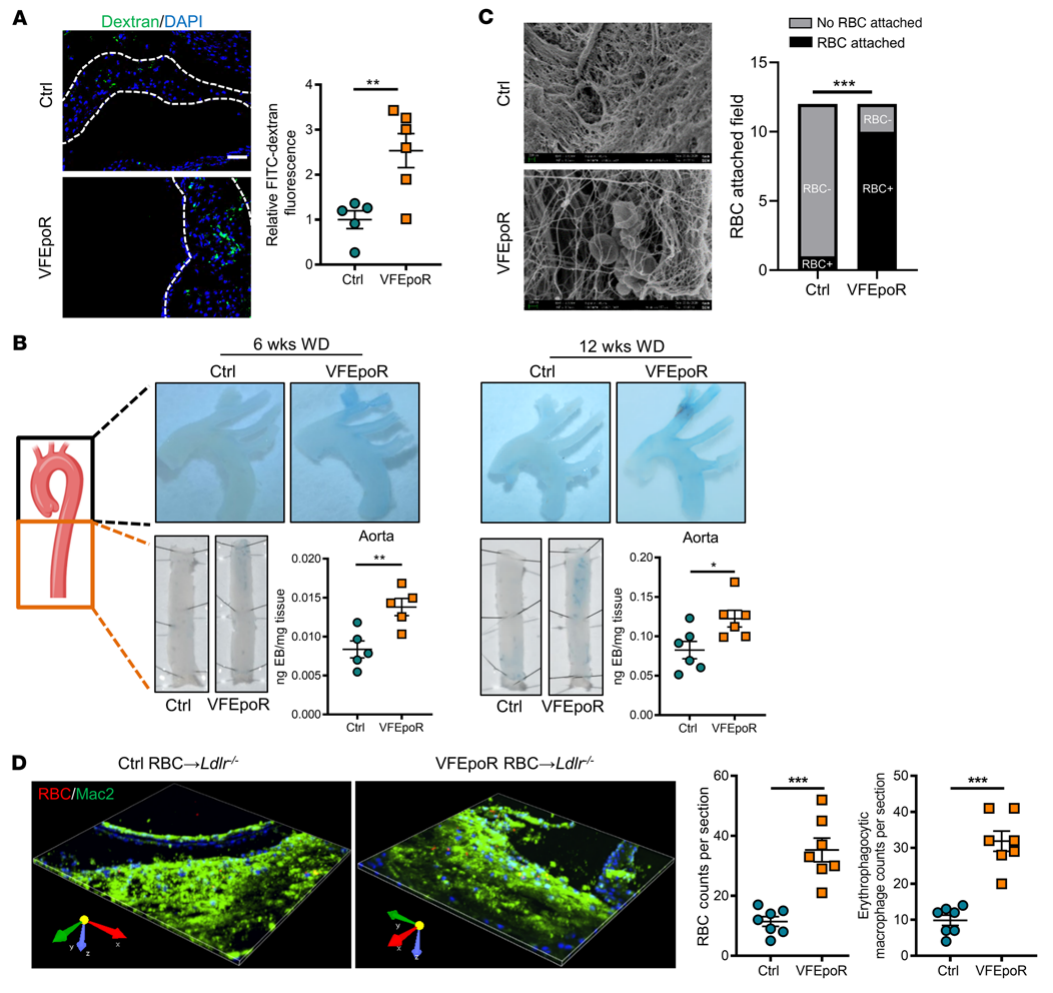
Aortic endothelial permeability was increased in VFEpoR mice. Control and VFEpoR mice were fed a Western diet (WD) and treated with LDLR ASO and EPO (3 times per week) for 6 or 12 weeks. (**A**) FITC dextran (green, 70 kDa) and nuclei (blue, DAPI) in aortic root cross-section, which were probed for endothelial permeability after 12 weeks of a WD. Scale bar: 50 μL. (**B**) Aortic arch and descending aorta were probed for endothelial permeability using Evans blue intravital staining after 6 or 12 weeks of a WD. The bar graph shows quantification of Evans blue extravasation normalized by the tissue weight. (**C**) En face scanning electron microscopy showing the luminal surface of the aortic arches; the bar graph shows numbers of the field that had RBC attachment or no RBC attachment (*n =* 3 per group); χ^2^ test. (**D**) *Ldlr^–/–^* mice were fed a WD for 2 weeks and then transfused with packed and PKH26-labeled 80–100 μL control or VFEpoR RBCs once per week for another 5 weeks, in total 7 weeks of WD. Representative 3D-rendered image from RBC-transfused mice aortic root lesions staining of macrophages (anti-Mac2, green) and RBCs (PKH26, red), and quantification of infiltrated RBC counts and erythrophagocytic macrophage counts in the lesions. Size: 332.80 μm × 332.80 μm × 7.50 μm. Calibration: XY:0.65 μm, Z:1.50 μm. Unpaired 2-tailed *t* test. *P < 0.05, **P < 0.01.

The increased entry of multiple cell types into atherosclerotic lesions suggested disruption of arterial endothelial barrier function. The uptake of hemoglobin by CD163^+^ macrophages has been shown to increase intraplaque angiogenesis in very advanced atherosclerotic lesions, with increased neovascular permeability (42). However, assessment of Von Willebrand factor staining did not show increased lesional angiogenesis in VFEpoR mice (Supplemental Figure 3E). Intravenous administration of 70 kDa FITC-dextran followed by high-resolution imaging of the aortic root revealed increased lesional FITC-dextran deposition in VFEpoR mice (Figure 4A). In addition, intravascular administration of albumin complexed with Evans blue showed more intense staining in the aortic arch and descending aorta in VFEpoR mice fed the WD for 12 weeks (Figure 4B). Increased Evans blue staining was also observed after feeding the WD for only 6 weeks, prior to lesion formation, indicating that increased permeability was not secondary to increased lesion formation in VFEpoR mice (Figure 4B). Hemodynamic stress could induce breaches in the arterial intima, leading to RBC infiltration and increased atherogenesis (43). We assessed this possibility by scanning electron microscopy with VFEpoR and littermate controls fed the WD for 12 weeks. This showed marked endothelial injury, with prominent loss of endothelial cells in VFEpoR mice (Figure 4C). These aortic denudations were associated with local RBC clusters and sometimes platelets (Figure 4C). Such phenotypes were rare in littermate EpoR-Cre control mice with low-dose EPO treatment (Figure 4C). To determine whether endothelial damage was associated with increased entry of RBCs into atherosclerotic lesions, we labeled control and VFEpoR RBCs with a membrane-integrating dye (PKH26) (Supplemental Figure 3F), and then transfused them in *Ldlr^–/–^* mice fed a WD diet for 2 weeks, followed by injection once per week for another 5 weeks to similar levels (Supplemental Figure 3G). 3D confocal imaging of aortic root lesions showed increased RBC infiltration and increased erythrophagocytosis of VFEpoR RBCs compared with WT RBCs (Figure 4D).

### Erythrophagocytosis of VFEpoR RBCs promotes macrophage ferroptosis.

**Figure 5:**
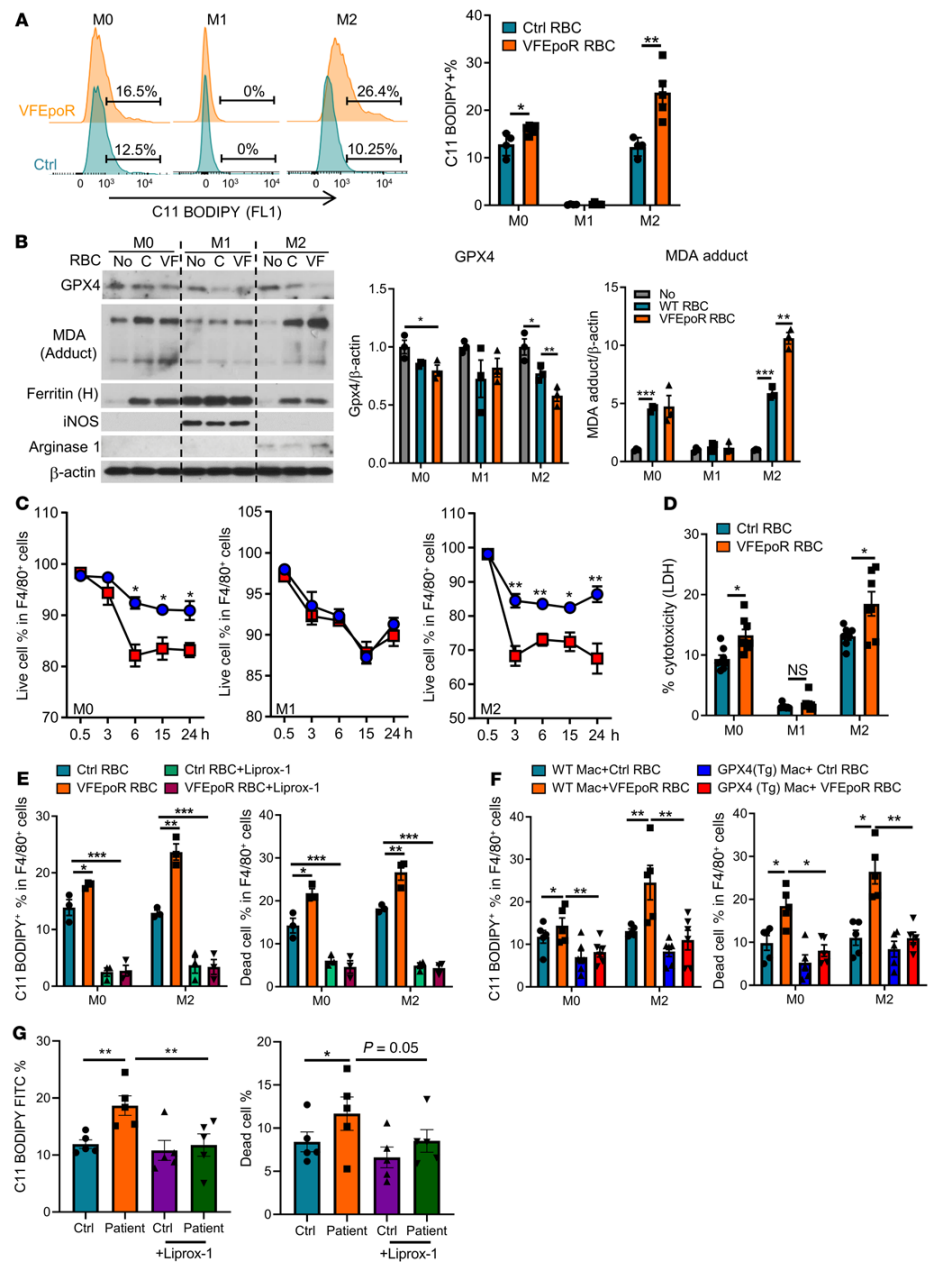
Macrophage lipid peroxidation and ferroptosis induced by erythrophagocytosis are reversed by Liprox-1. WT BM-derived macrophages were treated with vehicle (M0), LPS plus IFN-γ (M1), or IL-4 (M2) for 24 hours and then incubated with an equal number of control or VFEpoR RBCs for another 6 hours. (**A**) Representative C11 BODIPY histogram and statistics of C11 BODIPY^+^ macrophage percentage by flow cytometry. (**B**) Immunoblot of glutathione peroxidase 4 (GPX4), malondialdehyde modified proteins (MDA adduct), ferritin, arginase I, and inducible NOS (iNOS) and β-actin of M0, M1, and M2 cell lysates. The bar graph shows quantification of immunoblots normalized to β-actin. C denotes control and VF denotes VFEpoR RBCs. (**C**) M0, M1, and M2 macrophage viability were quantified as the percentage of propidium iodide–negative macrophage (live cell) versus total macrophages by flow cytometry (*n =* 3 replicates). (**D**) Macrophage LDH release in culture medium was measured after incubation with control or VFEpoR RBCs for 6 hours. (**E**) C11 BODIPY^+^ macrophage percentage and cell death ratio were tested by flow cytometry after 6 hours of erythrophagocytosis assay with or without Liprox-1 (200 nM) treatment. (**F**) M0 or M2 BM-derived macrophages from WT or GPX4 transgenic mice were treated with control and VFEpoR RBCs for 6 hours. Lipid peroxidation and cell death were assessed by C11-BODIPY and propidium iodide staining and analyzed by flow cytometry. (**G**) Human peripheral monocyte-derived macrophages generated from healthy donors were treated overnight with RBCs from JAK2^VF^-positive patients with MPN or matched healthy controls in the presence or absence of Liprox-1 (200 nM). Lipid peroxidation and cell death were assessed by C11-BODIPY and propidium iodide staining and analyzed by flow cytometry; unpaired 2-tailed *t* test (**A**, **C**, and **D**) or 1-way ANOVA (**B** and **E**–**G**). *P < 0.05, **P < 0.01, ***P < 0.001.

The evidence of lipid peroxidation in RBCs, macrophage death in association with increased staining for ferroptosis marker TfR, and necrotic core formation led us to further investigate the role of macrophage ferroptosis in atherogenesis in VFEpoR mice. Ferroptosis is a distinct mode of cell death induced by the accumulation of iron and lipid peroxides that is defined by the cellular response to specific chemical activators or inhibitors in the absence of evidence for other modes of cell death (44). Moreover, ferroptosis is prominently induced by defects in glutathione metabolism, including glutathione peroxidase 4 (GPX4) deficiency (44). To assess a potential role of ferroptosis, we incubated RBCs from control and VFEpoR mice with WT BM-derived macrophages (BMDMs). In order to increase the uptake of RBCs by phagocytosis, we treated macrophages with IL-4, which produced M2 macrophages, and compared their response to untreated (M0) or cytokine treated (M1) macrophages. Control and VFEpoR RBCs showed similar rates of erythrophagocytosis (Supplemental Figure 4A). After incubation with the same number of VFEpoR or littermate control RBCs for 6 hours, cellular lipid peroxidation as assessed by C11-BODIPY staining was increased in M0 and M2 but not M1 macrophages (Figure 5A), consistent with a report that M1 macrophages are resistant to ferroptosis (45). Phagocytosis of VFEpoR RBCs caused decreased GPX4 and increased formation of the lipid peroxidation end product malondialdehyde in M2 macrophages (Figure 5B). VFEpoR or control RBC uptake by macrophages caused a comparable increase in the iron-binding protein ferritin (Figure 5B).

Cell death, assessed by propidium iodide staining and flow cytometry, was increased in M2 and M0 but not M1 macrophages after phagocytosis of VFEpoR RBCs (Figure 5C). This was confirmed by more pronounced lactate dehydrogenase release from M0 and M2 macrophages in response to VFEpoR RBC phagocytosis (Figure 5D), consistent with increased ferroptotic cell death. We also treated cells with the ferroptosis inhibitor Liprox-1 that inhibits lipid peroxidation by functioning as a reactive radical-trapping antioxidant (46). Liprox-1 markedly reduced M0 and M2 cell death induced by phagocytosis of VFEpoR or control RBCs, abolishing the difference between genotypes (Figure 5E). The reduced cell death was associated with a marked reduction of cellular lipid peroxidation (Figure 5E). Moreover, transgenic overexpression of GPX4 (47) in macrophages (Supplemental Figure 4B) completely reversed the increased lipid peroxidation and macrophage cell death caused by VFEpoR RBCs (Figure 5F). In contrast, supplementation of GPX4 substrate glutathione ethyl ester (GSH-EE), ROS inhibitor N-acetyl cysteine (NAC), or inhibitors of other forms of cell death, including Z-VAD-FMK (pan-caspase/apoptosis/pyroptosis inhibitor) and necrostatin-1 (necroptosis inhibitor), failed to rescue erythrophagocytosis-induced lipid peroxidation and cell death (Supplemental Figure 4, C and D). Moreover, caspase 1/11–deficient macrophages showed similar levels of cell death as WT macrophages in response to VFEpoR RBCs (Supplemental Figure 4E), excluding a role of inflammasome-mediated caspase activation and pyroptosis in cell death. These results confirmed lipid peroxidation and ferroptosis as the likely cause of erythrophagocytosis-induced cell death.

To assess human relevance, we evaluated monocyte-derived, macrophage-mediated phagocytosis of control or JAK2^VF^ RBCs from patients with polycythemia vera. Erythrophagocytosis of JAK2^VF^ RBCs by WT macrophages increased macrophage lipid peroxidation and cell death compared with control RBCs, and both effects of JAK2^VF^ RBCs were reversed by Liprox-1 (Figure 5G).

### Liprox-1 reverses increased atherosclerosis in VFEpoR mice.

**Figure 6:**
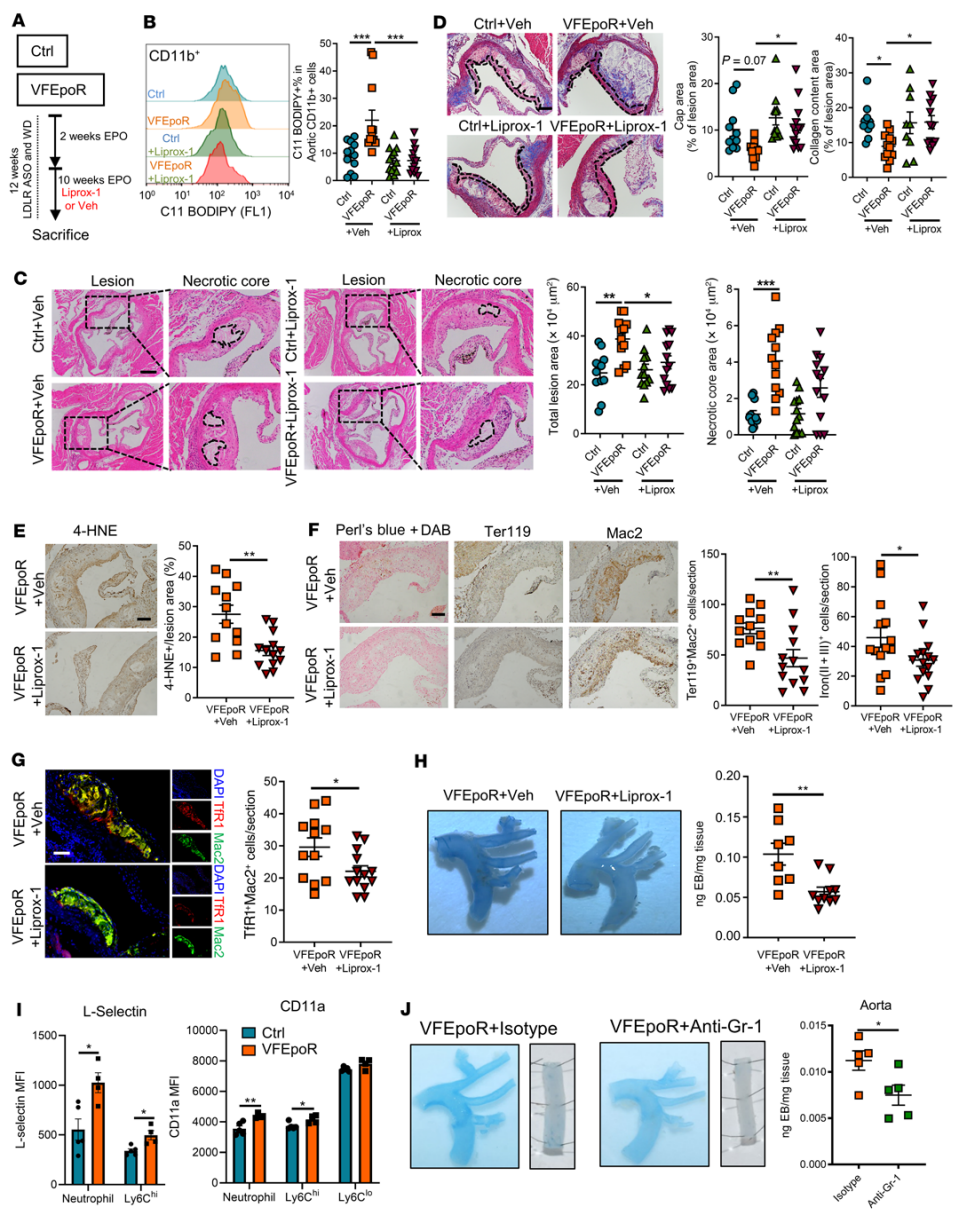
Liprox-1 reverses VFEpoR-induced atherosclerosis progression. Control and VFEpoR mice were fed a Western diet and treated with LDLR ASO and EPO for 12 weeks, with Liprox-1 (10 mg/kg, 3 times per week) or vehicle injection for the last 10 weeks. (**A**) Experiment timeline. (**B**) Representative C11 BODIPY staining histogram and quantification of C11 BODIPY CD11b^+^ cells as the percentage of total aortic CD11b^+^ cells. (**C**) H&E-stained images of aortic root sections. Necrotic core regions indicated by broken lines and quantification of total lesion area and necrotic core area are shown. Scale bar: 200 μm. (**D**) Aortic root sections were stained with Masson’s trichrome staining for fibrous cap (red, outlined by broken lines) and collagen (blue) content area, and then quantified as the ratio of total lesion area. Scale bar: 100 μm. (**E**) 4-HNE IHC in the aortic root cross sections. Scale bar: 100 μm. (**F**) Representative images of Perl’s blue plus DAB staining, IHC staining of RBCs (anti-Ter119), and macrophages (anti-Mac2) in aortic roots. Bar graph shows quantification of iron (II + III)–positive and erythrophagocytosis (Ter119^+^Mac2^+^) cell counts per section. Scale bar: 100 μm. (**G**) Immunofluorescence staining of TfR1 and macrophage (anti-Mac2), and quantification of TfR1 and macrophage costaining cell counts per section. Scale bar: 50 μm. (**H**) Evans blue intravital staining of arches and quantification of Evans blue extravasation. (**I**) L-selectin and CD11a expression levels in neutrophils and monocytes from peripheral blood assessed by flow cytometry. (**J**) VFEpoR mice were fed a Western diet and injected with LDLR ASO and EPO for 3 weeks, then divided into two groups, treated with Isotype or anti–Gr-1 mAb twice per week for another 4 weeks. In total 7 weeks WD and EPO injection. Evans blue intravital staining of arches and descending aorta, and quantification of extravasation. One-way ANOVA (**B**–**D**) or unpaired 2-tailed *t* test (**E**–**J**). *P < 0.05, **P < 0.01, ***P < 0.001.

To assess the role of ferroptosis in plaque formation in VFEpoR mice, we treated VFEpoR and littermate control mice with Liprox-1 or vehicle for 10 weeks (Figure 6A). Plasma total cholesterol, body weight, and blood cell counts were unaltered by Liprox-1 (Supplemental Figure 5, A–C). Liprox-1 reversed increased cellular lipid peroxidation in aortic CD11b^+^ myeloid cells in VFEpoR mice while having no effect in littermate controls (Figure 6B). Liprox-1 completely reversed the increase in aortic root lesion area, largely reversed the increased necrotic core area, and augmented fibrous cap area and collagen content in VFEpoR mice while having no impact in controls (Figure 6, C and D). Liprox-1 decreased lesional 4-HNE staining, iron deposits, RBC fragment accumulation, erythrophagocytosis, and macrophage TfR staining (Figure 6, E–G). Liprox-1 also decreased endothelial permeability in VFEpoR mice (Figure 6H).

We carried out further experiments to determine the mechanism by which Liprox-1 reduced endothelial permeability. Lipid peroxidation in VFEpoR splenic CD11b^+^ cells was reduced by Liprox-1 (Supplemental Figure 5D). ROS levels were selectively increased in circulating neutrophils and in Ly6C^hi^ but not Ly6C^lo^ monocytes in VFEpoR mice, and this increased ROS was reversed by Liprox-1 (Supplemental Figure 5E). However, Liprox-1 did not affect ROS levels in RBCs, neither in VFEpoR nor in littermate controls (Supplemental Figure 5F). The finding that Liprox-1 decreased endothelial permeability in association with reduced lipid peroxidation or ROS levels in leukocytes but not RBCs suggests a major role of leukocytes in the disruption of the endothelial barrier integrity in this setting. There was increased expression of adhesion molecules L-selectin and CD11a in circulating neutrophils and Ly6C^hi^ monocytes in VFEpoR mice (Figure 6I), which could mediate increased binding to endothelial cells. Depletion of leukocytes positive for granulocyte receptor-1 (Gr-1), including neutrophils and monocytes by anti-Gr-1 antibody, markedly reduced endothelial permeability in VFEpoR mice (Figure 6J and Supplemental Figure 3H). This suggests a major role of WBCs containing elevated ROS and lipid peroxidation products in mediating endothelial damage in VFEpoR mice.

There is evidence that lipid peroxidation drives gasdermin D–mediated cell death in lethal polymicrobial sepsis (48). Thus, we bred VFEpoR mice with mice deficient in gasdermin D, the common mediator of pyroptotic cell death downstream of inflammasome activation (48, 49). Gasdermin D deficiency had no effect on total lesion or necrotic core area (Supplemental Figure 5G) in VFEpoR mice. We also assessed levels of phosphorylated mixed-lineage kinase domain-like (p-MLKL), a marker of necroptosis (50), in lesional cells and showed no difference between VFEpoR and control mice (Supplemental Figure 5H). These in vivo results confirmed that ferroptosis is likely the major form of regulated cell death that contributes to atherogenesis in VFEpoR mice.

### Liprox-1 alleviates atherogenesis in Jak2^VF^ clonal hematopoiesis.

**Figure 7:**
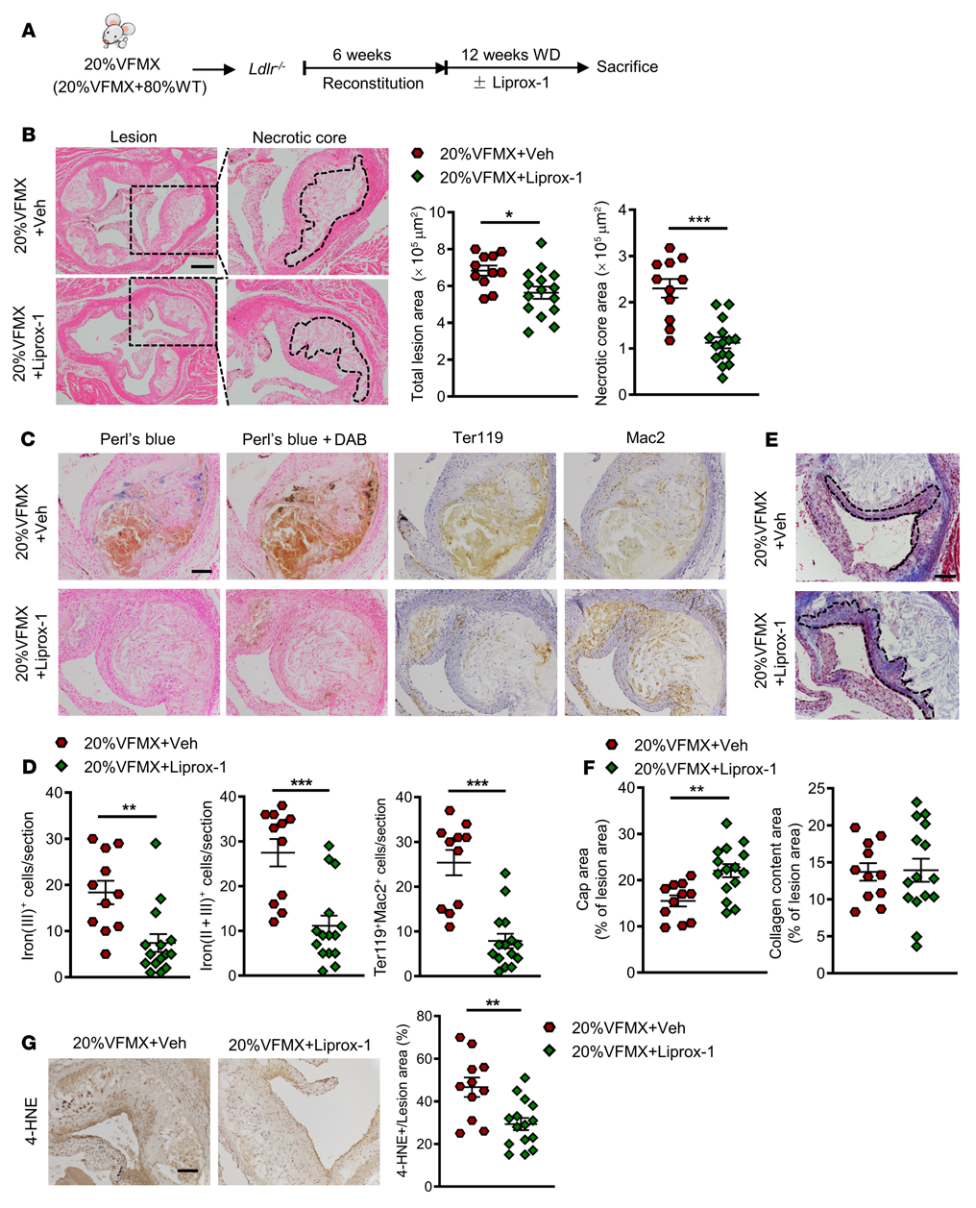
Liprox-1 alleviated accelerated atherosclerosis progression in *Jak2^VF^Mx-Cre* clonal hematopoiesis. 20% of *Jak2^VF^MX-Cre* (VFMX) mice were fed with Western diet together with Liprox-1 (10mg/kg, 3 times per week) or vehicle injection for 12 weeks. (**A**) Experiment timeline. (**B**) H&E-stained images of aortic root sections. Necrotic core regions indicated by broken lines, and quantification of total lesion area and necrotic core area are shown. Scale bar: 200 μm. (**C**) Iron (Perl’s blue) and redox-active iron deposition (Perl’s blue + DAB); IHC staining of RBCs (anti-Ter119) and macrophages (anti-Mac2) in aortic roots. (**D**) Bar graph shows quantification of iron (II + III)–positive and erythrophagocytosis (Ter119^+^Mac2^+^) cell counts per section. Scale bar: 100 μm. (**E**) Aortic root sections were stained with Masson’s trichrome staining for fibrous cap (red, outlined by broken lines) and collagen (blue) content area, and then (**F**) quantified as the ratio of total lesion area. Scale bar: 100 μm. (**G**) Lipid peroxidation product 4-HNE staining, quantified as the percentage of total lesion area. Scale bar: 100 μm. Unpaired 2-tailed *t* test. *P < 0.05, **P < 0.01, ***P < 0.001.

We next assessed the impact of ferroptosis inhibition on atherogenesis in *Jak2^VF^* mice simulating human *JAK2^VF^* clonal hematopoiesis without EPO supplementation. We generated *Ldlr^–/–^* mice with chimeric *Jak2^VF^* expression by transplanting a mixture of 20% *Jak2^VF^* BM cells from MX1-Cre/*Jak2^VF^* mice and 80% BM cells from WT mice, as previously reported (15). The recipients were fed a WD diet for 12 weeks with or without Liprox-1 (Figure 7A). The vehicle-treated *Jak2^VF^* chimeric mice showed marked atherosclerosis with pronounced necrotic cores, as previously reported (15), in association with prominent lesional iron deposition, erythrophagocytosis, and lipid peroxidation (Figure 7, C, D, and G). Liprox-1 markedly reduced lesion area, decreased the necrotic core, and increased fibrous cap area (Figure 7, B, E, and F) without altered plasma cholesterol, blood cell counts, spleen weight, body weight, or plasma IL-18 levels (Supplemental Figure 6, A–E). Lesional iron deposition, erythrophagocytosis, and lipid peroxidation were also reduced by Liprox-1 (Figure 7, C and D). These data indicate that RBC-related ferroptosis promoted atherogenesis in *Jak2^VF^* clonal hematopoiesis. Since mice transplanted with 20% *Jak2^VF^* cells have some features of MPN, such as splenomegaly, they can be considered to model clonal hematopoiesis but do not fit the definition of clonal hematopoiesis of indeterminate potential (CHIP) (51).

## Discussion

Our studies provide the first direct evidence for a causal role of *Jak2^VF^* erythroid lineage expression in exacerbated atherosclerosis. Mechanistically, increased RBC lipid peroxidation linked to reduced expression of glutathione-restoring proteins and increased entry of RBCs into plaques through damaged endothelium lead to macrophage erythrophagocytosis and ferroptosis. Endothelial damage appeared to be mediated not by RBCs but rather by neutrophils and monocytes containing increased ROS and lipid peroxidation products. The reversal of RBC-exacerbated atherosclerosis by the ferroptosis inhibitor Liprox-1 suggests a potential treatment to reduce CVD risk in JAK2-mutant myeloproliferative disorders, in patients with *JAK2^VF^* clonal hematopoiesis, and even more broadly in patients with elevated hematocrit and coronary artery disease.

Macrophage death is a central event contributing to formation of plaques with increased necrotic cores, an important cause of plaque instability (52). Macrophage death secondary to increased free cholesterol–mediated ER stress and apoptosis that overwhelms efferocytotic capacity has been proposed as a key mechanism promoting necrotic core formation (53–55). In addition, other forms of cell death, including pyroptosis secondary to inflammasome activation and necroptosis, can contribute to necrotic core formation (56–59). Heme has been reported as a potent stimulator of macrophage NLRP3 inflammasome activation and pyroptosis (60). However, pan-caspase inhibition or caspase 1/11 deficiency did not reverse erythrophagocytosis-induced cell death, excluding this mechanism. Recently, our group showed that macrophage-specific expression of *Jak2^VF^* promotes atherogenesis and features of plaque stability through AIM2 inflammasome activation and gasdermin D cleavage (15, 22). In contrast, in the present study, we found that *Jak2^VF^* RBC phagocytosis by WT macrophages induced gasdermin D–independent ferroptotic cell death.

Ferroptosis is a mode of regulated cell death resulting from the peroxidation of phospholipids containing long chain polyunsaturated fatty acids (44). Iron has a key role in lipid peroxidation and ferroptosis (44). However, the similar erythrophagocytosis rate of VFEpoR and control RBCs and a comparable increase in the iron-binding protein ferritin in macrophages do not support RBC-derived iron as a major cause of increased lipid peroxidation in macrophages. Rather, ferroptosis is likely induced by the uptake of VFEpoR RBCs that contain increased amounts of ROS and lipid hydroperoxides that seed macrophages for further ROS and lipid peroxide generation and reduce GPX4 levels. The inhibition of this process by Liprox-1 is consistent with its known mechanism of action as a radical-trapping antioxidant, which protect lipids from autoxidation and the autocatalytic radical chain reaction that produces lipid hydroperoxides (46). The increase in RBC lipid peroxides and ROS is related to increased NOX2 expression and decreased expression of several key enzymes in glutathione metabolism uncovered by our proteomics analysis. Defects in glutathione formation, regeneration, or reductive activity on lipid hydroperoxides are key features of ferroptosis (44).

Our studies suggest that both direct and indirect effects of Liprox-1 ameliorate atherosclerotic plaque formation. Liprox-1 reduced lipid peroxidation and staining for the ferroptosis marker TfR in plaques, indicating a direct effect within plaques. Liprox-1 also reversed the increase in aortic permeability in VFEpoR mice and reduced RBC infiltration without changing hematocrit or RBC ROS. Although *Jak2^VF^* was not expressed in endothelium or in myeloid cells, splenic red pulp macrophages, neutrophils, and monocytes showed increased lipid peroxides, suggesting that ingestion of *Jak2^VF^* RBCs by WBCs in the spleen or other sites led to increased lipid peroxide formation, consistent with the role of splenic red pulp macrophages and Ly6C^hi^ monocytes in erythrophagocytosis (23). Splenic WBC production contributes significantly to circulating and plaque WBCs (61). Liprox-1 selectively reversed increased ROS in circulating Ly6C^hi^ monocytes and neutrophils in VFEpoR mice. Since increased ROS in circulating leukocytes cause endothelial injury (62), this may explain why Liprox-1 treatment prevented increased endothelial permeability and entry of RBCs into plaques. This scenario is also supported by increased expression of integrins that would promote WBC binding to the endothelium in VFEpoR mice, even though WBCs did not express *Jak2^VF^* and WBC counts were not increased, and by reduced endothelial permeability when Gr-1^+^ leukocytes were depleted in VFEpoR mice.

In contrast to our findings in control hyperlipidemic mice, a recent study by Bai et al. (63) reported reduced atherosclerosis in *Apoe^–/–^* mice treated with the ferroptosis inhibitor ferrostatin-1, perhaps suggesting a broader role of ferroptosis in murine atherosclerosis. However, ferrostatin treatment was associated with reduced LDL and LDL oxidation, and a specific role of RBC infiltration and ferroptosis was not established. Moreover, RBCs are not usually prominent in atherosclerotic lesions of *Ldlr^–/–^* or *Apoe^–/–^* mice.

Our study has several translational and therapeutic implications. Inhibitors of lipid peroxidation and ferroptosis could have a role in preventing the atherothrombotic complications of *Jak2^VF^*-associated clonal hematopoiesis or polycythemia vera. Control of hematocrit in polycythemia vera via phlebotomy effectively reduces atherothrombotic risk (64). However, many patients have difficulty obtaining hematocrit goals by phlebotomy alone and may require additional treatments, such as the cytotoxic agent hydroxyurea or the JAK1/2 inhibitor ruxolitinib (65). Similar JAK1/2 inhibitors have recently received an FDA safety warning based in part on possibly increased coronary artery disease and thrombotic risk (66).

Beyond *Jak2^VF^*-accelerated atherothrombosis, there may be broader applications of ferroptosis inhibitors in RBC-promoted atherosclerosis. In a mouse atherosclerosis model with disturbed blood flow, RBC accumulation was associated with microscopic breaches in the endothelium and increased expression of VCAM-1, suggesting that endothelial disruption permits entry of RBCs and leukocytes into lesions, promoting vascular inflammation; these changes recapitulate findings in human coronary atherosclerosis (43, 67). RBC infiltration and macrophage erythrophagocytosis are associated with lesional lipid and protein oxidation, even at early stages of human atheroma (5). Notably, in our study, Liprox-1 markedly reduced ferroptotic cell death in WT macrophages ingesting control RBCs. Thus, erythrophagocytosis and ferroptosis may promote advanced atherosclerosis in humans with intraplaque hemorrhage (3, 68, 69) or endothelial barrier breach (43) and erosion (70), and thus patients with established coronary atherosclerosis might also benefit from ferroptosis inhibition.

Finally, ferroptosis is being intensely investigated in the realm of cancer, with genetic evasion of ferroptosis worsening some malignancies and ferroptosis inducers ameliorating cancer progression in a variety of different models (71). Our studies raise a concern that such agents could have adverse effects by promoting features of atherosclerotic plaque instability.

## Methods

### Mice.

*Jak2^V617F^* mice were created and reported previously (72); all results described pertain to *Jak2^V617F^* germline–expressing heterozygous mice. EpoR-Cre mice were originally generated by Achim Heinrich et al. (73). Gasdermin D knockout mice were obtained from Genentech (49). GPX4 transgenic mice were provided by Holly Van Remmen (Oklahoma Medical Research Foundation; ref. 47). All mice were housed under a 12-hour light/12-hour dark cycle and bred in a pathogen-free condition. Where indicated, EPO (Genscript, Z02975) was injected 3 times per week or every other day at a dose of 0.5 U/g. In the Liprox-1 treatment cohort, Liprox-1 was dissolved with 5% DMSO. Control and VFEpoR mice or *Jak2^VF^* clonal hematopoiesis mice were i.p. injected 3 times per week with Liprox-1 (10 mg/kg) or vehicle (5% DMSO in saline). To create hypercholesterolemia, GalNAc-conjugated Gen 2.5 ASO targeting mouse low-density lipoprotein receptor provided by Ionis Pharmaceuticals was used. The mice were i.p. injected with LDLR ASO once a week at a dose of 5 mg/kg body weight for the indicated time. This study used 6- to 9-week-old female mice.

### Study participants.

The JAK2^V617F^-positive patients with MPN were newly identified, untreated women (*n* = 3) and men (*n* = 2) from 38 to 57 years old. Sex-, age-, and ethnic group–matched healthy individuals were used as the controls. Blood was taken from the vein and used for RBC preparation.

### Atherosclerosis lesion analysis and metabolic profiling.

The aorta was cleaned and exposed under a binocular microscope. Pictures of the aortic arch and brachiocephalic artery were taken using a camera fitted to the binocular microscope. The aortic root from the heart was embedded in paraffin and then serially sectioned. Six sections per mouse were stained with H&E for total lesion and necrotic core area quantification. Smooth muscle fiber and collagen content staining were performed using Masson’s trichrome staining kit (Sigma-Aldrich, HT15) following the manufacturer’s instructions. Total plasma cholesterol was measured using kits from WAKO Diagnostics. Plasma EPO was tested using a commercial kit (BioLegend, 442707).

### Complete blood count.

Complete blood counts were performed using whole blood collected from facial vein bleeding in EDTA-coated tubes and then analyzed with Forcyte Veterinary Hematology Analyzer (Oxford Science, Inc.).

### Erythrocyte protein isolation.

First, 100 μL whole blood was collected from facial vein bleeding and centrifuged at 200*g* for 3 minutes to get rid of platelets and buffy coat, and then RBCs were washed with PBS; this step was repeated 3 times. RBC pellet was resuspended using PBS and filtered with Acrodisc PSF syringe filter to get rid of WBCs (Pall, DN AP4851). Cell lysates were incubated with protein A/G beads with rotation for 1 hour at 4˚C. After centrifugation at 11,773*g* for 10 minutes, the supernatant was collected and protein concentration was tested. We used approximately 50 μg protein/sample, mixed with 2-mercaptoethanol in an SDS sample buffer, and then heated the samples at 95°C for 5 minutes for Western blot. The supernatant was also used for the measurement of reduced GSH and oxidized glutathione (GSSG) ratio in the red blood cells using a commercial kit (Abcam, ab205811).

### RBC proteomics sample preparation.

The blood from at least 3 mice were pulled for proteomics. For RBC lysis, hemoglobin removal, and on-bead digestion, RBCs (400 million cells per sample) were diluted with Milli-Q. water with protease inhibitor cocktail to a final volume of 215 μL, mixed, incubated for 2 hours at –20°C, and thawed to room temperature. The lysate was separated from cell debris by centrifugation (22 min, 4°C, 16,000*g*) and 190 μL of supernatant was transferred to pre-wettened Hemovoid beads (30 mg loaded on supplied membrane insert in the 1.5 mL tube). The hemoglobin removal was performed based on the protocol in the Hemovoid kit with mixing for 5 minutes on a thermal shaker (VWR, 1500 rpm, 24°C) and centrifugation at 2400*g*. The sample was mixed with Hemovoid binding buffer (1:1) and washed 3 times with Hemovoid washing buffer (HVWB, 250 μL). After a last wash, 100 mM DTT in HVWB was added to the beads (final concentration 10 mM), mixed for 10 minutes at room temperature, and incubated for 30 minutes at 60°C. Samples were cooled and 200 mM iodoacetamide (IAA) in HVWB was added (final concentration 10 mM) and incubated for 45 minutes at room temperature in darkness with shaking and then centrifuged (5 min, 15,000*g*). Samples were first digested with LysC (MS grade, Pierce) in HVWB with protease/protein ratio 1:100 overnight at 37°C and then with trypsin (MS grade, VWR) in HVWB with protease/protein ratio 1:30. The peptide mixture was eluted to the next protein low binding tube and the digestion was stopped by formic acid (FA, final concentration 5%). Peptide concentration was determined by Pierce BCA peptide kit. An aliquot of 10 μg of peptides was desalted with C18 spin columns (Pierce), dried, and resuspended in 0.1% FA and 2% acetonitrile (ACN) prior to analysis.

### Liquid chromatography tandem mass spectrometry data acquisition and extraction.

Liquid chromatography tandem mass spectrometry (LC-MS/MS) analyses were performed using an UltiMate 3000 Nano UHPLC System connected to Q Exactive HF mass spectrometer (Thermo Fisher Scientific). For each sample, approximately 2 μg protein was injected from a cooled autosampler onto 50 cm long EASY-Spray HPLC column (2 μm C18 particles, 75 μm × 500 mm, Thermo Fisher Scientific). The chromatographic separation was achieved using a gradient of 90 minutes from 4% to 26% of solvent B (98% ACN/0.1% FA). Full mass spectra were acquired with a resolution of R = 120,000 (at 200 *m/z*), followed by up to 17 consecutive data-dependent MS/MS spectra taken using higher-energy dissociation with 28 normalized collisional energy. Samples were analyzed in a randomized order. MS/MS data were extracted and processed in Proteome Discoverer v2.4 (Thermo Fisher Scientific) and searched against the UniProt complete proteome database (55,466 mouse protein sequences).

### RBC preparation, PKH26 labeling, and RBC transfusion.

Whole blood was collected from control and VFEpoR mice in 10% citrate phosphate dextrose adenine anticoagulant solution. After centrifuging at 300*g* for 10 minutes, the buffy coat was removed. RBCs were further leuko-reduced using a murine-adapted Pall Acrodisc PSF 25 mm WBC filter or a Pall neonatal filter with Leukosorb media. For the PKH26 labeling, 1 × 10^8^ RBCs were resuspended in 1 mL dilute C, and then mixed with another 1 mL dilute C containing 4 μM PKH26 (Sigma-Aldrich, MIDI26). After incubating at room temperature for 5 minutes, staining was stopped by 0.5% BSA. After washing 2 times with PBS, following centrifugation, packed RBCs were diluted 1:1 to 1:4 with sterile PBS. Diluted RBCs (80–100 μL, hemoglobin range from 17 to 17.5 g/dl) (74) were i.v. transfused into *Ldlr^–/–^* recipient mice within 2 hours after preparation.

### Immunofluorescence staining and IHC.

For the immunofluorescence staining, paraffin-embedded slides were deparaffinized and rehydrated in Trilogy (Cell Marque, 920P-09). Identification of macrophages, NETosis, proliferation, and endothelial cells in atherosclerotic lesions were performed by immunostaining using anti-Mac-2 (Cedarlane, CL8942AP, 1:10,000), biotinylated myeloperoxidase (MPO) (R&D Systems, BAF3667, 1:30), anti-histone H3 antibody (Abcam, ab5103, 1:300), anti-Ki67(Abcam, ab15580, 1:100), anti-vWF (Abcam, ab117113, 1:100), anti–p-MLKL (Abcam, 196436, 1:100), and anti-TfR (Thermo Fisher Scientific, 13-6800, 1:100). The sections were incubated with primary antibodies overnight at 4˚C and then incubated with secondary antibodies for 30 minutes. TUNEL staining was performed using a commercially available kit (Roche, 12156792910). For the TfR staining, a M.O.M kit was used (Vector Laboratories, BMK-2202) following the manufacturer’s instructions. Sections were mounted using ProLong Gold Antifade Mountant with DAPI (Thermo Fisher Scientific, P3693) and imaged using a Leica DMI6000B microscope. For the confocal microscopy imaging, sections were incubated with primary antibodies, including anti-Mac-2, anti-Ter119 (Thermo Fisher Scientific, 13-5921-82, 1:200), and anti-ACTA2 Cy3 (Sigma-Aldrich, C6198 1:500). Sections were mounted using DAPI. Imaging was conducted on a Zeiss LSM 880 NLO confocal microscope with a 60× oil lens. *Z*-stack images were acquired with an average of 5 slices and analyzed using FIJI/ImageJ software.

For the IHC staining, secondary anti-rat IgG and anti-rabbit IgG (Vector Laboratories, MP-7405) were incubated with sections for 30 minutes. The reaction was developed with DAB staining (Vector Laboratories, SK-4100). The iron staining (Abcam, ab150674) was performed using a commercially available kit.

In all immunofluorescence or IHC staining, isotype-matched normal IgG was used as the negative control.

### Flow cytometry.

Flow cytometry to characterize peripheral blood neutrophils, monocytes, or BM hematopoietic stem and progenitor cell profiles was performed as previously described (22). For the ROS measurement, RBCs or lysed WBCs were labeled with APC/Cy7 LIVE/DEAD (Invitrogen, MP34955) and antibodies APC anti-Ter119 (BioLegend, 116212), anti-CD45 (BioLegend, 103128), PE-Cy7 anti-CD11b (BioLegend, 101216), BV421 anti–Gr-1 (BioLegend, 108445), and APC anti-CD115 (BioLegend, 134410) in staining buffer for 20 minutes at 4°C, and then washed once with staining buffer and resuspended in 10 μM H_2_ DCFDA (Thermo Fisher Scientific, 88-5930-74) for another 15 minutes at 37°C. For the lipid peroxidation assay, after labeling, the cells were resuspended in 2 μM C11-BODIPY (Thermo Fisher Scientific, D3861) for 30 minutes at 37°C. For circulating leukocyte adhesion marker detection, PE anti-CD11a (BioLegend, 101107) and L-selectin (BioLegend, 104406) were used. Flow cytometry was performed using the LSR Canto or LSRII (Becton Dickinson) and data were analyzed using FlowJo software (Becton Dickinson). Isotype-matched normal IgG was used as the control in each flow cytometry assay.

### Erythrophagocytosis assays.

BMDMs were prepared as described (22). For erythrophagocytosis assays, macrophages were plated in 12-well culture plates. Refrigerator-stored 7–9 days erythrocytes from control and VFEpoR mice were added (macrophage/erythrocyte ratio was about 1:10) into macrophages. Following 6 hours of incubation, the macrophages were thoroughly washed with PBS 3 times and then analyzed by flow cytometry or Western blot.

For erythrophagocytosis assay using human RBCs, peripheral blood samples were obtained from patients with JAK2^V617F^ or matched healthy controls. RBC-rich fractions were obtained with High Efficiency Leukocyte Reduction Filter (Haemonetics Corporation, NEO1). Human peripheral monocyte-derived macrophages generated from healthy donors were used for the assays and plated in 24-well, no-tissue culture plates as previously reported (22). RBCs were added and incubated overnight (macrophage/RBC ratio was about 1:10). After washing for 3 times with PBS to remove the free RBCs, the RBCs were labeled with PerCP/cyanine 5.5 anti-CD235a (BioLegend, 349110) and macrophages were stained with APC anti-CD68 (BioLegend, 333810). Lipid peroxidation was measured by the C11-BODIPY, and cell death was determined by propidium iodide staining.

### Immunoblotting.

Macrophages were lysed in RIPA buffer containing protease inhibitor on the ice for 10 minutes and then centrifuged at 14,000*g* for 5 minutes to generate protein lysates. The protein concentration was determined by BCA assays and then mixed with 4× Laemmli sample buffer and heated at 95°C for 5 minutes. Protein was separated by 4%–20% gradient SDS-PAGE and transferred onto nitrocellulose membranes. Then, the membranes were blocked with 5% nonfat milk in TBST and immunostained with primary antibodies, anti-GPX4 (Novus, MAB5457, 1:1000) anti-ferritin (Abcam, ab65080, 1:3000), anti-malondialdehyde (Abcam, ab6463, 1:2000), anti-arginase 1 (Cell Signaling Technology, 93668s, 1:1000), and β-actin (Cell Signaling Technology, 4970s, 1:5000) at 4°C overnight and detected using HRP-conjugated secondary antibodies.

### Real-time quantitative PCR assay for JAK2V617F.

The amplification and detection were performed on an ABI Prism 7000 analyzer (Applied Biosystems) with an initial step of 10 minutes at 95°C, followed by 40 cycles of 15 seconds at 95°C and 1 minute at 60°C. The following PCR primers and probes were used: JAK2WT, forward, GTCAGCTTTCTCACAAGCATTT, probe, TGGTGTCTGTGTCTG; TGGAGAGGA, reverse, CTTCAGGTATGTATCCAGTGATCC; JAK2V617F, forward, GTCAGCTTTCTCACAAGCATTT, probe, TGGTGTCTGTTTCTGTGGAGA; GGAGA, reverse, CTTCAGGTATGTATCCAGTGATCC.

### Endothelial permeability assays.

Evans blue (0.5%, 200 μL, Sigma-Aldrich, E2129) and dextran (70 kDa, 10 mg/mL, Sigma-Aldrich, 46945) were i.v. injected. After 1 hour, the mice were euthanized and the heart and aorta were carefully dissected under a microscope. For the Evans blue, the descending aorta and arch were separated and recorded with a microscope camera. For the mice receiving dextran injection mice, the aortic roots were embedded in OCT and serial sections were obtained by cryosectioning the frozen aortic roots. The sections were fixed in acetone for 5 minutes and washed in PBS 4 times. Sections were mounted using ProLong Gold Antifade Mountant with DAPI (Thermo Fisher Scientific, P3693) and imaged using a Leica DMI6000B microscope.

### Depletion of Gr-1^+^ myeloid cells.

Mice received i.p. injections of isotype control (BioXcell, BE0090, 250 μg/mouse) and anti–Gr-1 antibody (BioXcell, BE0075, 250 μg/mouse) twice per week. The successful depletion after antibody injection was verified by flow cytometry.

### Statistics.

Data that passed the normality test were analyzed using 2-tailed Student’s *t* test for 2 groups; 1-way ANOVA with Tukey’s post hoc analysis for more than 2 groups; or 2-way ANOVA with Šidák’s post hoc analysis for 2 factors. Data that were not normally distributed were analyzed using the nonparametric Mann-Whitney *U* test, or, for more than 2 groups, a Kruskal-Wallis test with post hoc analysis using Dunn’s test. Outliers were removed using Grubb’s test (GraphPad Prism). Data are presented as mean ± SEM. A *P* value less than 0.05 was considered as a significant difference. Statistical analyses were conducted and analyzed using GraphPad Prism.

### Study approval.

Written consent was obtained from each patient before participation in this study and was approved by the First Hospital of Jilin University. All protocols were approved by the IACUC of Columbia University.

## Author contributions

WL, NÖ, MY, HD, KEU, YT, XH, TX, TPF, and SA performed research and analyzed data. WL, YGY, OS, ART, and NW designed research, analyzed data, or wrote the paper.

## Supplementary Material

Supplemental data

## Version 1 05/19/2022

In-Press Preview

## Version 2 07/01/2022

Electronic publication
