# Supplementary material for: Erythroid lineage Jak2V617F expression promotes atherosclerosis through erythrophagocytosis and macrophage ferroptosis
Source: J Clin Invest. 2022 Jul 1;132(13):e155724. doi: 10.1172/JCI155724 (PMC9246386; doi:10.1172/JCI155724)
Supplement: Supplemental data [file jci-132-155724-s113.pdf]

# Supplemental Figure 1

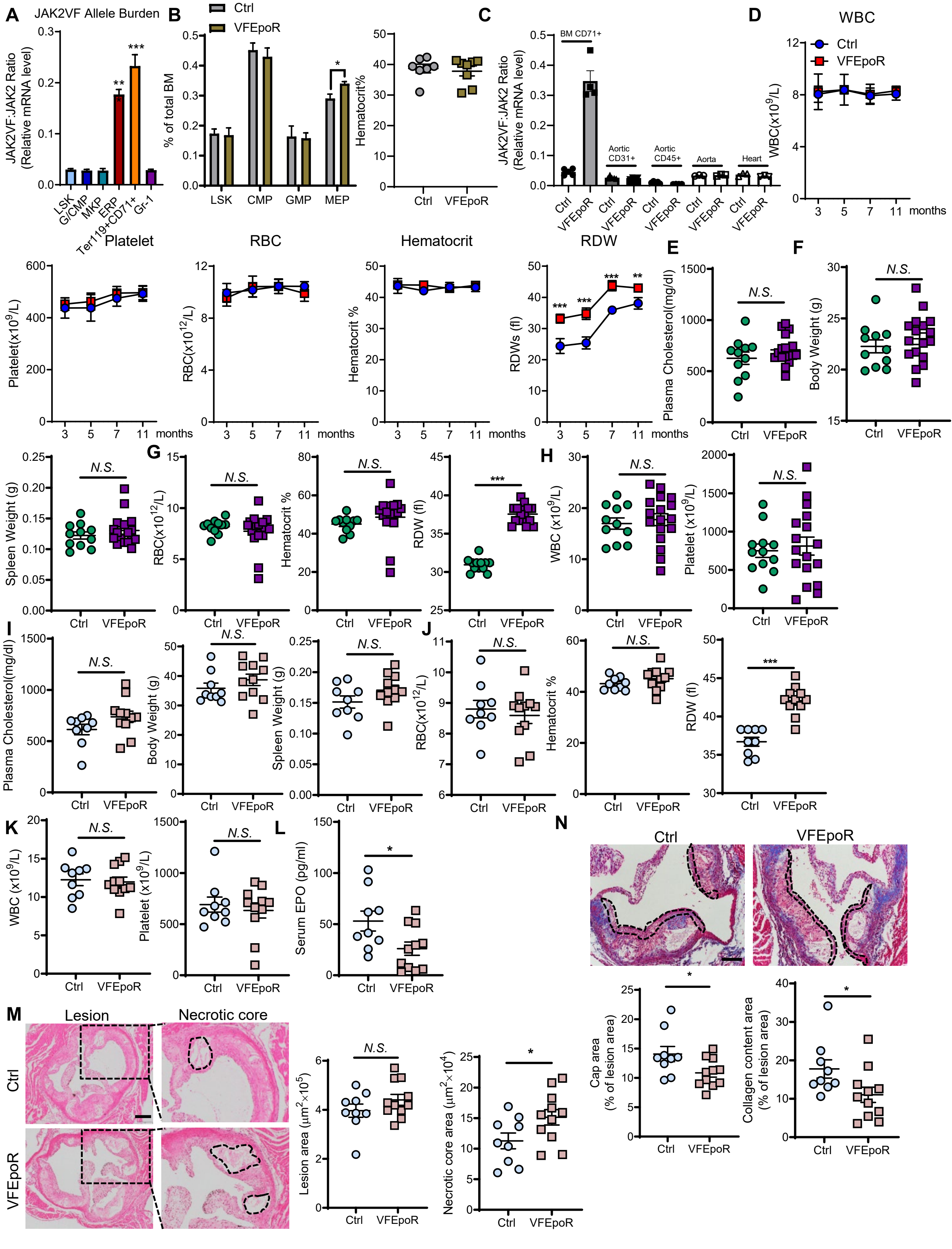

**Supplemental Figure 1:**. **A.** Quantification of allele-specific TaqMan qPCR of *Jak2$^{VF}$* to JAK2 ratio in progenitor and precursor cells. Progenitor cells were defined as LSK (Lin$^-$Sca1$^+$c-Kit$^+$), common myeloid progenitors (Lin$^-$Sca1$^-$c-Kit$^+$CD34$^{int}$FcγRII/III$^{int}$; CMP), granulocyte and macrophage progenitors (Lin$^-$Sca1$^-$c-Kit$^+$CD34$^{int}$FcγRII/III$^{hi}$; GMP), erythroid progenitor (Lin$^-$Sca1$^-$c-Kit$^+$CD34$^{int}$FcγRII/III$^{int}$CD71$^+$CD41$^-$, ERP) and Ter119$^+$CD71$^+$ and Gr-1 (Ly6C/Ly6G+). **B.** Bone marrow hematopoietic cell profile in VFEpoR mice, hematocrit and RDW of Ctrl and VFEpoR mice. **C.** Quantification of allele-specific TaqMan qPCR of *Jak2$^{VF}$* to JAK2 ratio in bone marrow erythroid lineage (CD71+), aortic endothelial cells (aortic CD31+), infiltrated white blood cells in aorta (aortic CD45+), whole aorta and whole heart. **D.** Peripheral white blood cell and platelet counts, RBC counts, hematocrit and RDW were monitored from 3 month to 11 month ages of ctrl and VFEpoR mice. n=5 mice per group. Ctrl and VFEpoR mice were fed with western diet and treated with LDLR ASO weekly for 12 weeks **(E-H) E.** Plasma total cholesterol level after 12 weeks western diet. **F.** Body weight and spleen weight. **G.** Peripheral blood RBC counts, hematocrit and RDW. **H.** White blood cell and platelet counts**.** Ctrl and VFEpoR mice were fed with western diet and treated with LDLR ASO weekly for 22 weeks (**I-N**). **I.** Plasma total cholesterol level after 22 weeks western diet, body weight and spleen weight. **J.** Peripheral blood RBC counts, hematocrit and RDW. **K.** White blood cell and platelet counts. **L.** Plasma EPO level. **M.** H&E staining and quantification of total lesion area and necrotic core area in the aortic root. Necrotic core regions are indicated by broken lines. Scale Bar, 100μm. **N.** Aortic root sections were stained with Masson trichrome staining for fibrous cap (red, outlined by broken lines) and collagen (blue) content area, and then quantified as the ratio of total lesion area. Scale Bar, 100μm. Unpaired 2 tailed *t* test. *$p$<0.05, **$p$<0.01, *** $p$<0.001.

# Supplemental Figure 2

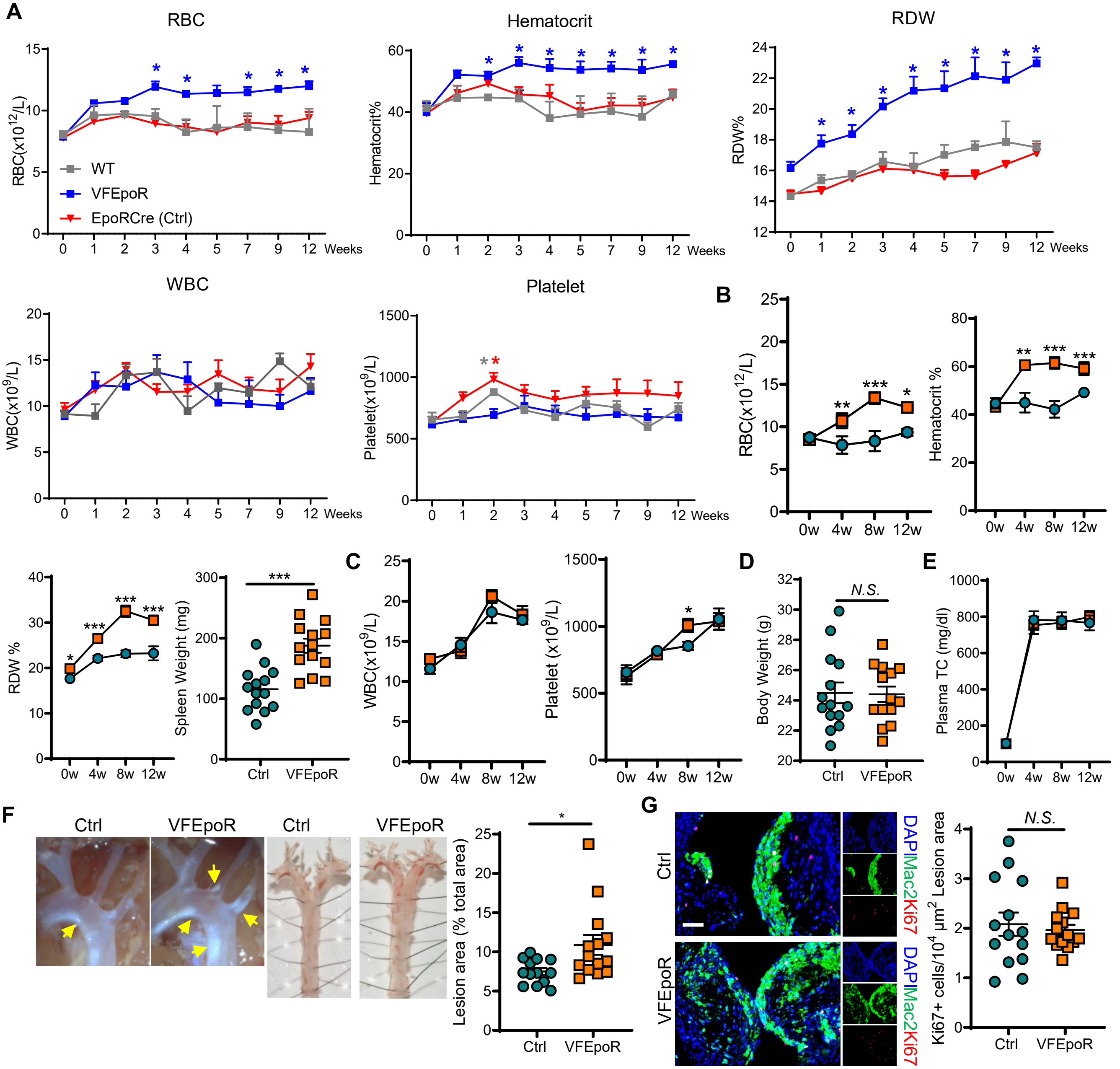

**Supplemental Figure 2: VFEpoR mice showed polycythemia vera phenotypes after EPO injection. A.** Ctrl, VFEpoR and WT mice were injected with 0.5U/g EPO 3 times per week for 12 weeks and fed with chow diet. Peripheral blood RBC counts, hematocrit, RDW, white blood cell and platelet counts were monitored during 12 weeks EPO injection. n=10 mice per group. Ctrl and VFEpoR mice were fed with western diet and treated with LDLR ASO and EPO (3 times per week) for 12 weeks (**B-G**). **B.** Peripheral RBC counts, hematocrit, RDW and spleen weight. **C.** White blood cell and platelet counts. **D.** Body weight. **E.** Plasma total cholesterol level. **F.** The plaque in aortic arches. The arrows indicate plaques; En face aortic plaque analysis with Oil red O staining and quantification of oil red O positive area as a percentage of total area. **G.** Representative images of aortic root sections in which proliferative cells were labeled by Ki67 (red) and macrophage by anti-Mac2 (green), counterstained with DAPI (blue) and quantification of normalized Ki67+ cells by lesion area. Scale Bar, 50μm. Unpaired 2 tailed *t* test. *$p$<0.05, **$p$<0.01, *** $p$<0.001.

**Supplemental Figure 3**

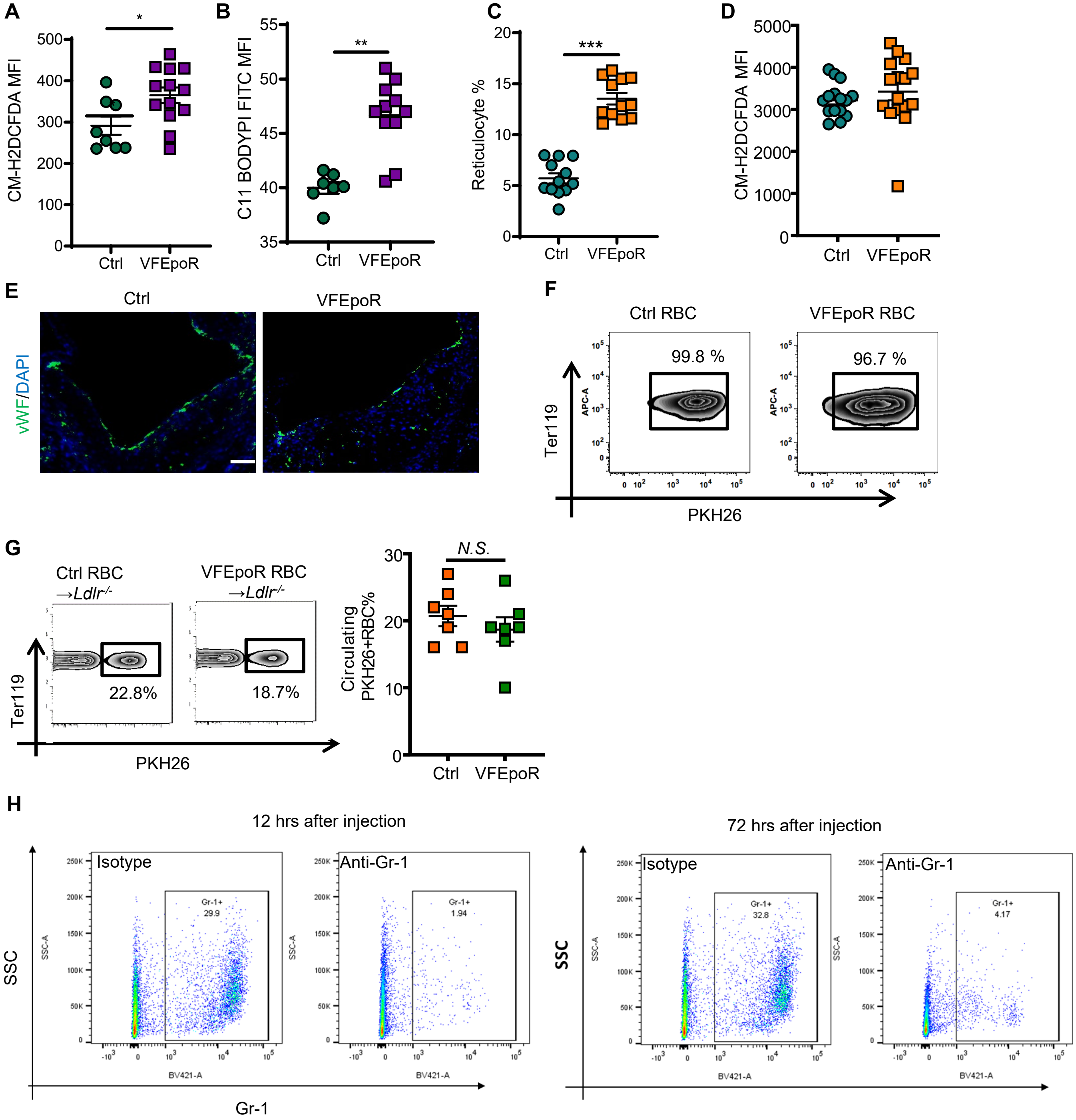

**Supplemental Figure 3. A.** ROS in RBCs was assessed by $H_2$DCFDA staining, and analyzed by flow cytometry as mean fluorescence intensity (MFI) in Ctrl and VFEpoR mice without EPO injection. **B.** RBC lipid peroxidation was assessed by C11 BODIPY staining and quantified by flow cytometry as MFI in Ctrl and VFEpoR mice without EPO injection. **C.** Reticulocyte percentage in blood quantified by flow cytometry. **D.** ROS in reticulocytes was assessed by $H_2$DCFDA staining, and quantified by flow cytometry as MFI. **E.** Representative immunofluorescence images of von Willebrand factor (green, vWF) and DAPI (Blue) in aortic root cross sections. Scale Bar, 50μm. **F.** Flow cytometry showed PKH26 RBC staining efficiency. **G.** Representative flow plots showing percentage of PKH26+ RBC in circulation after 5 weeks transfusion. **H.** Assessment of the efficiency of leukocytes depletion in mice treated with anti-Gr-1 mAb. Flow cytometric analysis of peripheral blood collected 12 hrs or 72 hrs after first injection. Unpaired *t* test. *** $p<0.001$.

**Supplemental Figure 4**

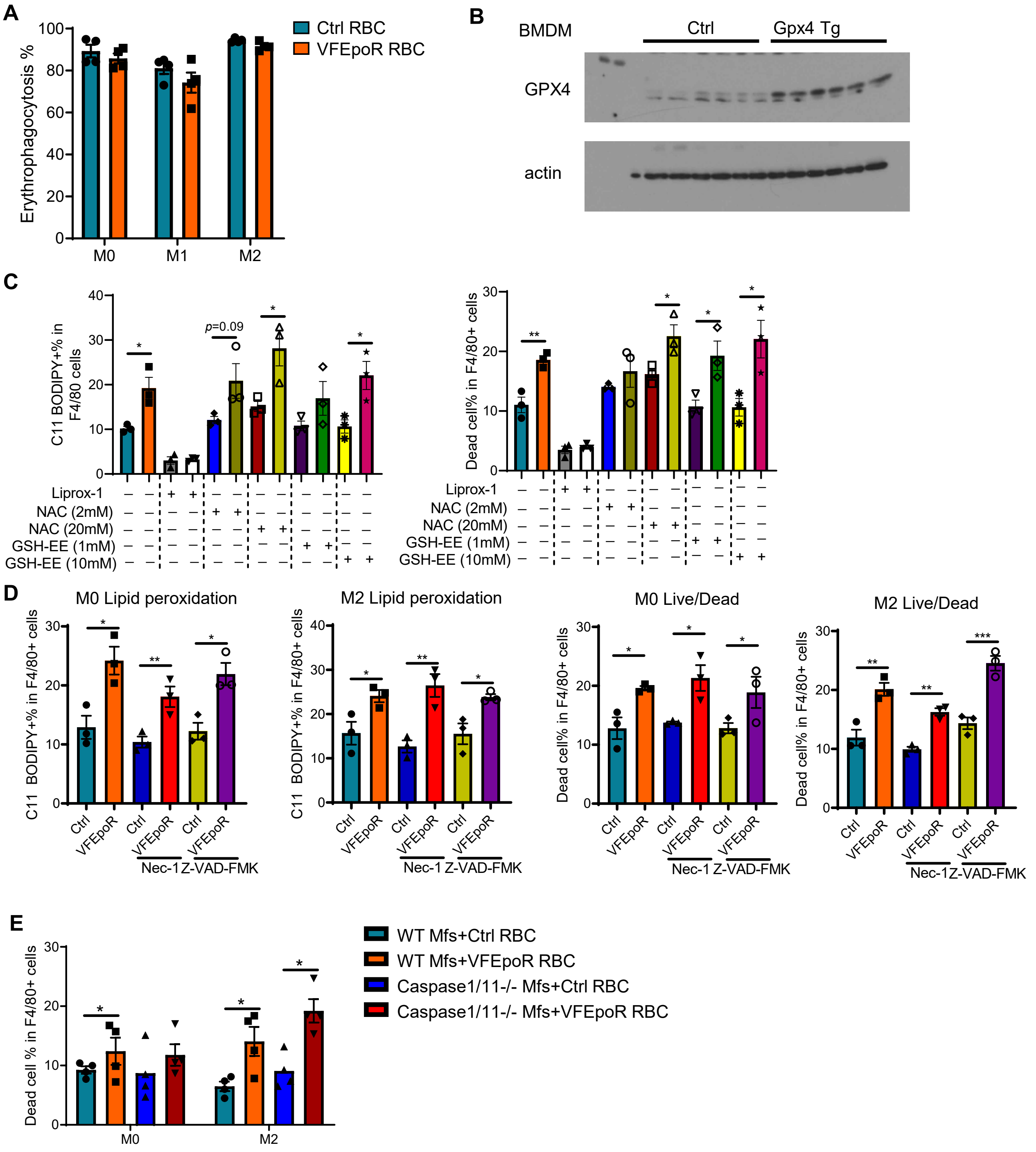

**Supplemental Figure 4. Erythrophagocytosis induced cell death is distinct from necroptosis and apoptosis and independent of caspase1/11. A.** Erythrophagocytosis rate in M0, M1 and M2 macrophage after Ctrl and VFEpoR RBCs 6hrs treatment. **B.** Western blot verification of GPX4 overexpression in the GPX4 transgenic mice bone marrow derived macrophage. **C.** WT bone marrow derived macrophages were treated with ROS inhibitor N-acetyl cysteine (NAC) in low (2mM) and high dose (20mM) or glutathione ethyl ester (GSH-EE) in low (1mM) and high dose (10mM). Macrophage lipid peroxidation and viability were assessed using C11 BODIPY and PI staining respectively, by flow cytometry. **D.** M0 and IL-4 induced M2 from WT bone marrow derived macrophages were treated with Ctrl and VFEpoR RBCs for 6 hours with or without Nec-1 (necroptosis inhibitor, 50μM) and Z-VAD-FMK (apoptosis inhibitor, 10μM). Macrophage lipid peroxidation and viability were assessed using C11 BODIPY and propidium iodide (PI) staining respectively, by flow cytometry. **E.** M0 or M2 bone marrow derived macrophages from WT or caspase1/11-/- mice were treated with Ctrl and VFEpoR RBCs for 6 hours. Macrophage viability was assessed using PI staining by flow cytometry. Unpaired 2 tailed *t* test (**A.**) or One-way ANOVA (**C.D.E**). *$p$<0.05, **$p$<0.01, *** $p$<0.001.

# Supplemental Figure 5

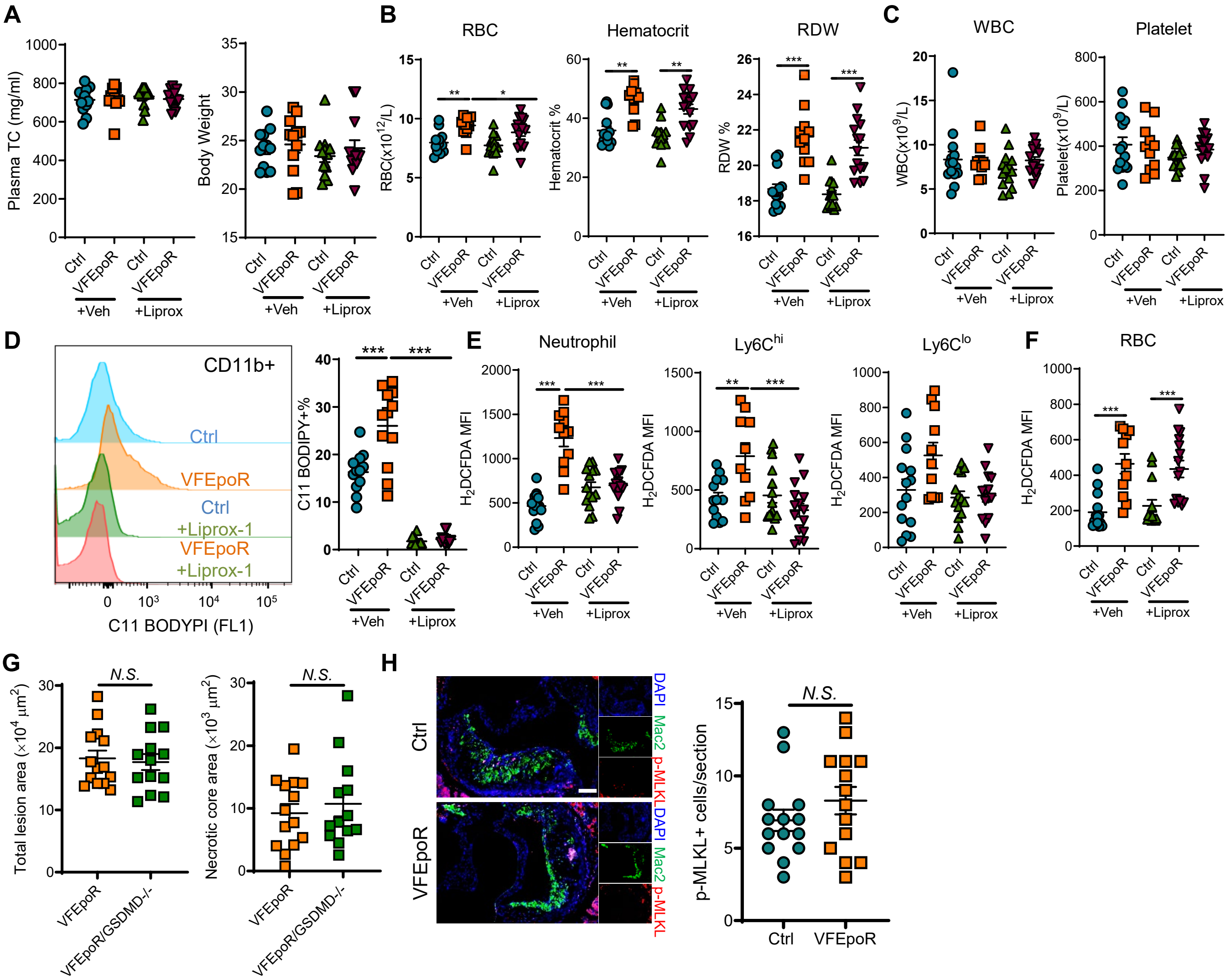

**Supplemental Figure 5. Liprox-1 reverses lipid peroxidation in splenic CD11b+ cells and increased ROS in circulating leukocytes but not RBCs in VFEpoR mice.** Ctrl and VFEpoR mice were fed with western diet and treated with LDLR ASO and EPO for 12 weeks, with Liproxstatin-1 (Liprox-1) or vehicle (10mg/kg, 3 times per week) injection for last 10 weeks. **A.** Plasma total cholesterol and body weight after 12 weeks western diet. **B-C.** Complete peripheral blood cell count of RBC counts, hematocrit and red blood cell distribution width, white blood cells and platelet counts after 12 weeks WD. **D**. Representative C11-BODIPY histogram in splenic CD11b+ cell and quantification of percentage of C11-BODIPY+ cells in splenic CD11b+ cells by flow cytometry. **E.** ROS levels in neutrophils and $Ly6C^{hi}$ or $Ly6C^{low}$ monocytes from peripheral blood as assessed by H2DCFDA staining. **F**. Quantification of ROS production in RBC by $H_2$DCFDA staining. MFI, median fluorescence intensity. **G.** VFEpoR and VFEpoR/$GSDMD^{-/-}$ mice were fed with western diet and treated with LDLR ASO and EPO (3 times per week) for 12 weeks, quantification of absolute total lesion area and necrotic core area in the aortic root cross sections. **H.** Representative images and quantification of p-MLKL (red) positive cells, macrophages by anti-Mac2 (green), and counterstained with DAPI (blue) in aortic root sections. Scale Bar, 75μm. Unpaired 2 tailed *t* test (**G.H.**) or One-way ANOVA (**A.B.C.D.E.F.**), ***$p$<0.001. *$p$<0.05, **$p$<0.01, *** $p$<0.001.

# Supplemental Figure 6

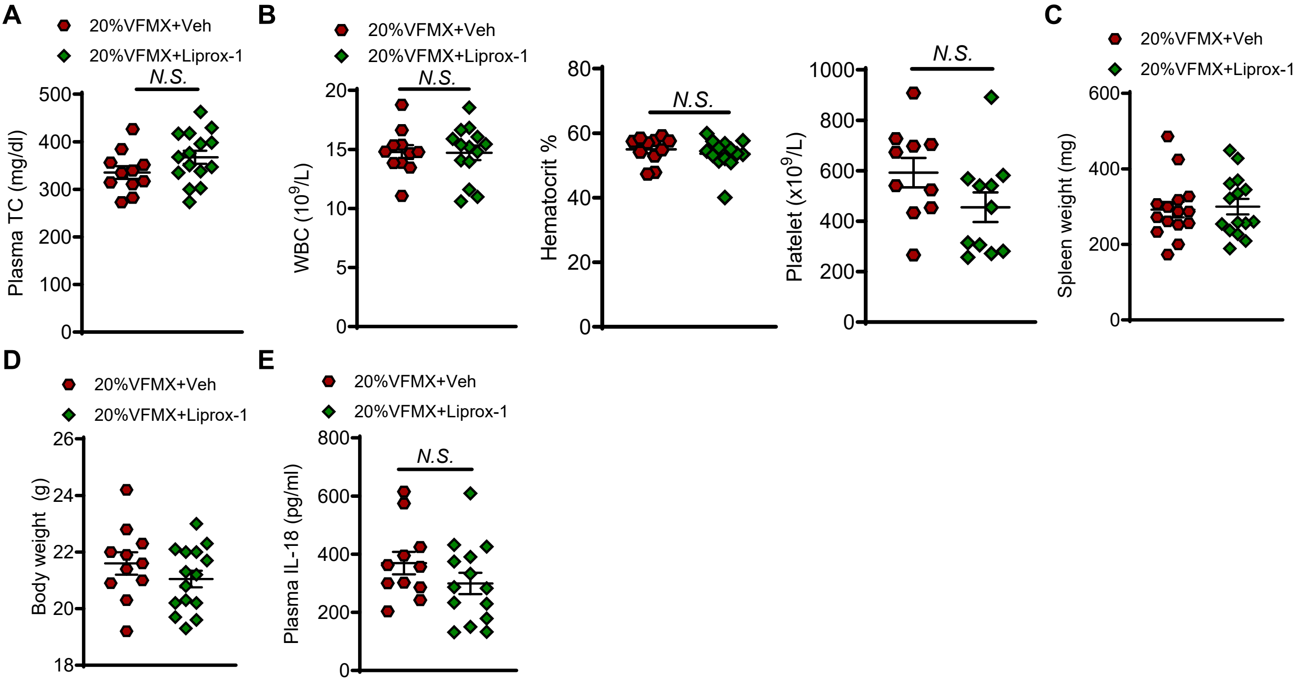

**Supplemental Figure 6.** 20% VFMX mice were fed with western diet together with Liproxstatin-1 (10mg/kg, 3 times per week) or vehicle injection for 12 weeks. **A.** Plasma total cholesterol levels. **B.** Complete peripheral blood cell count of white blood counts, hematocrit and platelet counts. **C**. Spleen weight. **D.** Body weight. **E.** Plasma IL-18 levels. Unpaired 2 tailed *t* test.
